# Fatty acids in the de novo lipogenesis pathway and incidence of type 2 diabetes: A pooled analysis of prospective cohort studies

**DOI:** 10.1371/journal.pmed.1003102

**Published:** 2020-06-12

**Authors:** Fumiaki Imamura, Amanda M. Fretts, Matti Marklund, Andres V. Ardisson Korat, Wei-Sin Yang, Maria Lankinen, Waqas Qureshi, Catherine Helmer, Tzu-An Chen, Jyrki K. Virtanen, Kerry Wong, Julie K. Bassett, Rachel Murphy, Nathan Tintle, Chaoyu Ian Yu, Ingeborg A. Brouwer, Kuo-Liong Chien, Yun-yu Chen, Alexis C. Wood, Liana C. del Gobbo, Luc Djousse, Johanna M. Geleijnse, Graham G. Giles, Janette de Goede, Vilmundur Gudnason, William S. Harris, Allison Hodge, Frank Hu, InterAct Consortium, Albert Koulman, Markku Laakso, Lars Lind, Hung-Ju Lin, Barbara McKnight, Kalina Rajaobelina, Ulf Riserus, Jennifer G. Robinson, Cecilia Samieri, Mackenzie Senn, David S. Siscovick, Sabita S. Soedamah-Muthu, Nona Sotoodehnia, Qi Sun, Michael Y. Tsai, Tomi-Pekka Tuomainen, Matti Uusitupa, Lynne E. Wagenknecht, Nick J. Wareham, Jason H. Y. Wu, Renata Micha, Rozenn N. Lemaitre, Dariush Mozaffarian, Nita G. Forouhi

**Affiliations:** 1 MRC Epidemiology Unit, University of Cambridge, Cambridge, United Kingdom; 2 Cardiovascular Health Research Unit, Department of Epidemiology, University of Washington, Seattle, Washington, United States of America; 3 Department of Public Health and Caring Sciences, Clinical Nutrition and Metabolism, Uppsala University, Uppsala, Sweden; 4 The George Institute for Global Health, the Faculty of Medicine, University of New South Wales, Sydney, Australia; 5 Friedman School of Nutrition Science and Policy, Tufts University, Boston, Massachusetts, United States of America; 6 Department of Nutrition and Epidemiology, Harvard T.H. Chan School of Public Health, Boston, Massachusetts, United States of America; 7 Channing Division of Network Medicine, Department of Medicine, Brigham and Women’s Hospital and Harvard Medical School, Boston, Massachusetts, United States of America; 8 Institute of Epidemiology and Preventive Medicine, College of Public Health, National Taiwan University, Taipei City, the Republic of China; 9 Institute of Public Health and Clinical Nutrition, University of Eastern Finland, Kuopio, Finland; 10 Section of Cardiovascular Medicine, Department of Internal Medicine, Wake Forest University School of Medicine, Winston-Salem, North Carolina, United States of America; 11 INSERM, UMR 1219, Bordeaux Population Health Research Center, University of Bordeaux, Bordeaux, France; 12 USDA/ARS Children’s Nutrition Research Center, Department of Pediatrics, Baylor College of Medicine, Houston, Texas, United States of America; 13 Cancer Epidemiology Division, Cancer Council Victoria, Melbourne, Australia; 14 Centre of Excellence in Cancer Prevention, School of Population & Public Health, Faculty of Medicine, The University of British Columbia, Vancouver, Canada; 15 Department of Mathematics and Statistics, Dordt University, Sioux Center, Iowa, United States of America; 16 Department of Biostatistics, University of Washington School of Public Health, Seattle, Washington, United States of America; 17 Department of Health Sciences, Faculty of Science, Vrije Universiteit Amsterdam, Amsterdam Public Health Research Institute, Amsterdam, the Netherlands; 18 Division of Cardiology, Department of Medicine, Taipei Veterans General Hospital, Taipei City, the Republic of China; 19 Department of Medicine, Division of Cardiovascular Medicine, Stanford University School of Medicine, Stanford, California, United States of America; 20 Divisions of Aging, Department of Medicine, Brigham and Women's Hospital and Harvard Medical School, Boston, Massachusetts, United States of America; 21 Division of Human Nutrition and Health, Wageningen University, Wageningen, the Netherlands; 22 Centre for Epidemiology and Biostatistics, The University of Melbourne, Parkville, Australia; 23 Precision Medicine, School of Clinical Sciences at Monash Health, Monash University, Clayton, Australia; 24 Icelandic Heart Association Research Institute, Kopavogur, Iceland; 25 Department of Internal Medicine, Sanford School of Medicine, University of South Dakota, Sioux Falls, South Dakota, United States of America; 26 OmegaQuant Analytics, Sioux Falls, South Dakota, United States of America; 27 National Institute for Health Research Biomedical Research Centres Core Nutritional Biomarker Laboratory, University of Cambridge, Addenbrooke's Hospital, Cambridge, United Kingdom; 28 National Institute for Health Research Biomedical Research Centres Core Metabolomics and Lipidomics Laboratory, University of Cambridge, Addenbrooke's Hospital, Cambridge, United Kingdom; 29 Medical Research Council Elsie Widdowson Laboratory, Cambridge, United Kingdom; 30 Institute of Clinical Medicine, Internal Medicine, University of Eastern Finland, Kuopio, Finland; 31 Department of Medicine, Kuopio University Hospital, Kuopio, Finland; 32 Department of Medical Sciences, Uppsala University, Uppsala, Sweden; 33 Department of Internal Medicine, National Taiwan University Hospital, Taipei City, the Republic of China; 34 Preventive Intervention Center, Departments of Epidemiology, the University of Iowa College of Public Health, Iowa City, Iowa, United States of America; 35 The New York Academy of Medicine, New York, New York, United States of America; 36 Center of Research on Psychological and Somatic disorders, Department of Medical and Clinical Psychology, Tilburg University, Tilburg, the Netherlands; 37 Institute for Food, Nutrition and Health, University of Reading, Reading, United Kingdom; 38 Cardiovascular Health Research Unit, Department of Medicine, University of Washington, Seattle, Washington, United States of America; 39 Department of Laboratory Medicine and Pathology, University of Minnesota, Minneapolis, Minnesota, United States of America; 40 Public Health Sciences, Wake Forest School of Medicine, Winston-Salem, North Carolina, United States of America; Hangzhou Institute for Advanced Study, CHINA

## Abstract

**Background:**

De novo lipogenesis (DNL) is the primary metabolic pathway synthesizing fatty acids from carbohydrates, protein, or alcohol. Our aim was to examine associations of in vivo levels of selected fatty acids (16:0, 16:1n7, 18:0, 18:1n9) in DNL with incidence of type 2 diabetes (T2D).

**Methods and findings:**

Seventeen cohorts from 12 countries (7 from Europe, 7 from the United States, 1 from Australia, 1 from Taiwan; baseline years = 1970–1973 to 2006–2010) conducted harmonized individual-level analyses of associations of DNL-related fatty acids with incident T2D. In total, we evaluated 65,225 participants (mean ages = 52.3–75.5 years; % women = 20.4%–62.3% in 12 cohorts recruiting both sexes) and 15,383 incident cases of T2D over the 9-year follow-up on average. Cohort-specific association of each of 16:0, 16:1n7, 18:0, and 18:1n9 with incident T2D was estimated, adjusted for demographic factors, socioeconomic characteristics, alcohol, smoking, physical activity, dyslipidemia, hypertension, menopausal status, and adiposity. Cohort-specific associations were meta-analyzed with an inverse-variance-weighted approach. Each of the 4 fatty acids positively related to incident T2D. Relative risks (RRs) per cohort-specific range between midpoints of the top and bottom quintiles of fatty acid concentrations were 1.53 (1.41–1.66; *p* < 0.001) for 16:0, 1.40 (1.33–1.48; *p* < 0.001) for 16:1n-7, 1.14 (1.05–1.22; *p* = 0.001) for 18:0, and 1.16 (1.07–1.25; *p* < 0.001) for 18:1n9. Heterogeneity was seen across cohorts (I^2^ = 51.1%–73.1% for each fatty acid) but not explained by lipid fractions and global geographical regions. Further adjusted for triglycerides (and 16:0 when appropriate) to evaluate associations independent of overall DNL, the associations remained significant for 16:0, 16:1n7, and 18:0 but were attenuated for 18:1n9 (RR = 1.03, 95% confidence interval (CI) = 0.94–1.13). These findings had limitations in potential reverse causation and residual confounding by imprecisely measured or unmeasured factors.

**Conclusions:**

Concentrations of fatty acids in the DNL were positively associated with T2D incidence. Our findings support further work to investigate a possible role of DNL and individual fatty acids in the development of T2D.

## Abstract 2

Fumiaki Imamura and colleagues investigate the association between concentrations of fatty acid involved in de-novo lipogensis and incidence of Type 2 diabetes.

## Author summary

**Why was this study done?:**

- De novo lipogenesis (DNL) is a metabolic pathway involved in the endogenous synthesis of specific fatty acids, such as 16:0, 16:1n7, 18:0, and 18:1n9, and it is linked to the pathophysiology of cardiometabolic diseases, including type 2 diabetes (T2D).
- Circulating or tissue concentrations of these fatty acids have been investigated for the associations with T2D incidence in epidemiological research. However, published studies reported inconsistent associations inconsistently and were subject to publication bias.
- Summary evidence is not available to date for the associations between these fatty acids and T2D incidence. An integration of available cohort studies would increase statistical power and allow assessment of generalizability, standardization of analytical strategies, and evidence synthesis with the potential publication bias minimized.

**What did the researchers do and find?:**

- As a part of the Fatty Acids and Outcomes Research Consortium (FORCE), we conducted new individual-participant data analyses of 17 cohort studies of a total of 65,225 adults free of T2D at baseline, among whom 15,383 developed incident T2D over up to 20 years of follow-up.
- The cohort studies analyzed the associations between fatty acids (16:0, 16:1n7, 18:0, and 18:1n9) and the risk of developing T2D with standardized analytic strategy.
- In pooled analyses, each of the fatty acids was positively associated with a higher risk of developing T2D. The associations were independent of major risk factors for T2D, such as age, sex, race/ethnicity, socioeconomic characteristics, smoking status, physical activity, and obesity.

**What do these findings mean?:**

- The findings provide the first summary evidence to date for the positive relationships of concentrations of the DNL-related fatty acids with a risk of T2D, indicating the strong relevance of DNL and its determinants to the development of T2D.
- These fatty acids potentially reflect the status of DNL activity, which may be stimulated or suppressed by a combination of carbohydrate intake, alcohol intake, polyunsaturated fatty acid intake, and other lifestyle and clinical factors. Therefore, the current findings indicate the need for investigation into determinants and consequences of elevated concentrations of these fatty acids.
- Despite several advantages of our individual-level data analysis in this pooling project, the results cannot establish whether elevated concentrations of these fatty acids caused the development of T2D or whether underlying peripheral or hepatic insulin resistance, for example, may elevate both the fatty acid concentrations and the risk of T2D independently.

## Introduction

De novo lipogenesis (DNL) is a metabolic pathway for the endogenous synthesis of triglycerides and other lipids from dietary starch, sugar, and protein [1,2]. Palmitic acid (16:0) is the major fatty acid product of DNL and can be elongated to stearic acid (18:0) and desaturated to form palmitoleic acid (16:1n7) and from stearic acid to oleic acid (18:1n9). Tissue levels of these fatty acids have been previously reported to show associations with insulin resistance and to be higher among adults with type 2 diabetes (T2D) than healthy adults [3].

Experimental studies have supported causal detrimental effects of 16:0 on inflammatory responses and pancreatic function, whereas protective effects of 16:1n7 and 18:1n9 on pancreatic function have been suggested [4–7]. In addition, greater DNL activity has been reported to be driven by lifestyle habits such as excessive consumption of carbohydrates or alcohol and lower physical activity [8–10], although the relative contributions of different lifestyle habits influencing DNL remain undefined. Investigation into how fatty acids in the DNL pathway relate to incident T2D may provide important etiological knowledge and stimulate future work on modifiable risk factors and preventive treatments.

Individual studies have examined the associations between circulating DNL-related fatty acids and incident T2D, showing mixed associations [11–18]. For instance, higher concentrations of 16:0 were associated with a higher incidence of T2D in several studies but not in others [14,18]. Similarly inconsistent findings were observed for 16:1n7, 18:0, and 18:1n9. To our knowledge, no prior studies have comprehensively brought together available evidence relating these fatty acids to incidence of T2D or investigated potential factors underlying the heterogeneous findings. Varied findings to date could reflect unstable results from some relatively small-scale studies (e.g., *N* cases < 200 in many cohorts), different lipid fractions evaluated across studies, and differences in demographics and analytic approaches. Also, there is little evidence whether the fatty acids in the DNL pathway may have a pathophysiological role independent of the overall DNL activity or triglycerides, one of the end products of the DNL. Therefore, to better characterize the prospective associations of fatty acids in the DNL pathway with incidence of T2D, we conducted de novo pooled individual-level analysis using harmonized methods across 17 studies in the global Fatty Acids and Outcomes Research Consortium (FORCE).

## Methods

### Cohorts and study variables

FORCE was initially formed from the Cohorts for Heart and Aging Research in Genomic Epidemiology consortium. FORCE is an ongoing consortium project to study relationships of fatty acid biomarkers with health outcomes (http://force.nutrition.tufts.edu/) [19–21]. The current project included 17 prospective studies (cohorts and nested case-control or case-cohort studies). These studies agreed to participate after confirming the inclusion criteria met: recruitment of adults aged 18 years or over and without prevalent diabetes at the time of fatty acid assessment; available data of circulating or adipose 16:0, 16:1n7, 18:0, and 18:1n9; and ascertainment of incident T2D (S1 Text). Other cohorts participated in FORCE for other projects [19–21] but did not contribute to this study because incident T2D were not ascertained. All cohorts had obtained approval from each institutional review board and written informed consent from participants. This study is reported as per the Preferred Reporting Items for Systematic Reviews and Meta-Analyses (PRISMA) guideline (S1 Checklist).

A standardized analysis protocol was developed, approved by the FORCE investigators, and provided to each participating cohort (S2 Text). The protocol prespecified the inclusion criteria mentioned above, as well as the exposures (DNL-related fatty acids), standardized covariates, effect modifiers, incident T2D, and statistical methods. Following this protocol we developed centrally, each cohort performed new individual-participant data analysis. Cohort-specific results were recorded in a standardized electronic form and centrally compiled and meta-analyzed. The data underlying the results presented in the study are available for researchers who meet criteria of each participating cohort.

S1 Text includes information on participating cohorts, study participants, and methods for fatty acid measurement and ascertainment of incident T2D. Briefly, each cohort isolated fatty acid molecules from one or more lipid compartments including erythrocyte phospholipids, plasma phospholipids, plasma cholesteryl esters, plasma triglycerides, total plasma or serum, or adipose tissue. Then, in vivo fatty acid concentrations were measured with gas chromatography. Concentrations of each fatty acid were quantified as a percent of total fatty acids in the lipid fraction.

Incident T2D was ascertained on the basis of one or more criteria including fasting glucose ≥7.0 mmol/L; glucose 11.1 mmol/L from 2-hour oral glucose tolerance test; new use of oral antidiabetic medication; or concentration of HbA1c ≥ 6.5% (S1 Text). The Melbourne Collaborative Cohort Study (MCCS) [14] and Alpha Omega Cohort (AOC) [22] ascertained incident T2D based on self-reported physician diagnosis, use of antidiabetic medication, or both. The EPIC-InterAct Study ascertained incident T2D by adjudicating self-reported T2D diagnosis or verifying diagnosis in disease registries [17].

### Statistical analysis in individual studies

Individual-participant data analyses were prespecified and documented in the protocol, with the primary exposure variables being 16:0, 16:1n7, 18:0, and 18:1n9. We examined Pearson correlation coefficients between these fatty acids within each lipid fraction. To assess associations of interest, Cox proportional hazard regression was modeled to time-to-event data, with sampling weights applied in EPIC-InterAct with a case-cohort design [17]. Each cohort calculated follow-up time from time of fatty acid measurement to either date of incident T2D, death from any cause, or loss to follow-up, or censoring at end of follow-up, whichever available and occurred first. In the 2 cohorts (AOC and MCCS) without individuals’ person-time data [14,22], logistic regression was used as the most efficient approach to obtaining estimates of interest from the 2 cohorts. The fatty acid variables were evaluated as a continuous linear variable in a unit of the study-specific interquintile range (the difference between the midpoints of the top and bottom quintiles) and, in a separate model, as categorical indicator variables (quintile categories, with the lowest quintile as the reference). We used an interquintile range and quintile categories in continuous and categorical approaches, respectively, because two approaches allowed estimation of the associations over the same exposure range and improvement of comparability between the two approaches.

Covariates for statistical adjustment were prespecified, including their categorization (e.g., continuous, quintiles, etc.). Each participating study prespecified the use of some study-specific covariates (e.g., the number of categories for education status), depending on availability. The primary model included field site, age, sex, race/ethnicity, occupation, education, smoking status, physical activity, alcohol consumption, prevalent hypertension (self-reported or treated), prevalent dyslipidemia (self-reported or treated), prevalent heart disease, and self-reported health status. The second model further adjusted for adiposity measures (body mass index [BMI] and waist circumference). For the mechanistic investigation, the third model further adjusted for circulating 16:0 (for analysis of 16:1n7, 18:0, and 18:1n9) and triglycerides to assess whether associations of 16:1n7, 18:0, and 18:1n9 with incident T2D would be independent of 16:0 and triglycerides and for analysis of 16:0, of triglycerides.

We assessed study-specific measures of interaction by age, sex, BMI, and race/ethnicity using the second model that adjusted for potential confounders including the adiposity measures. Each fatty acid, these prespecified potential effect modifiers, and their relevant cross-product terms and variance-covariance measures were analyzed to evaluate the potential interaction within each cohort.

### Pooled analyses

Study-specific regression coefficients, either log hazard ratios or log odds ratios, and standard errors were meta-analyzed with an inverse-variance weighted method to estimate summary relative risks (RRs) and confidence intervals (CIs). Heterogeneity in results between studies was quantified as I^2^ [23]. A few cohorts included fatty acid measures in more than one lipid fraction. To avoid double-counting estimates from such cohorts, we prespecified primary use of estimates of phospholipid (plasma or erythrocyte) fatty acids. These lipid fractions were most commonly used among participating cohorts and generally reflect longer-term exposure than the other compartments except for adipose tissue [24]. In secondary analyses, estimates in each different lipid fraction were also meta-analyzed separately, using each available cohort with measurements in that lipid fraction.

To test interactions by age, sex, BMI, and race/ethnicity, cohort-specific coefficients of cross-product terms were meta-analyzed. Because we considered the tests for interactions as exploratory, we applied correction for multiple testing as α_two-tailed_ = 0.0031 (0.05/4 fatty acids/4 potential effect modifiers). If an interaction was statistically significant, stratum-specific associations were estimated by using regression coefficients and variance-covariance matrices and then pooled using meta-analysis. We fitted meta-regression models and stratified meta-analyses to investigate potential sources of heterogeneity due to study-specific characteristics. Factors examined included lipid fraction, geographical region (Europe/Australia, United States, Asia), and prevalence of dyslipidemia. To further explore sources of heterogeneity, we evaluated the following factors post hoc: prevalence of hypertension, mean triglyceride concentrations, fasting status, availability of time-to-event data, and mean years of follow-up. As a sensitivity analysis, we conducted random-effects meta-analysis and meta-analysis after converting odds ratios to risk ratios in AOC and MCCS [25]. Meta-analyses were performed using Stata 14.2 (StataCorp, College Station, Texas) with α_two-tailed_ = 0.05, unless specified otherwise.

## Results

### Population characteristics

Among 17 participating cohorts, mean age ranged from 52.3 to 76.0 years (Table 1). Three cohorts recruited men only, two cohorts recruited women only, and the others recruited both (%women = 20.4%–62.3%). Study-specific mean BMIs ranged from 25.2 to 28.4 kg/m^2^, except for the Chin-Shan Community Cardiovascular Cohort Study (CCCC) in Taiwan (mean BMI = 23.2 kg/m^2^). Most studies recruited participants of European descent predominantly. Participants of non-European descent were recruited in the Multi-Ethnic Study of Atherosclerosis (MESA; 71.6% nonwhite), the Women’s Health Initiative Memory Study (WHIMS; 11.6% nonwhite), the Cardiovascular Health Study (CHS; 11.0% nonwhite), and the CCCC (100% East Asian).

**Table 1:** Baseline characteristics of 17 studies of the pooling analysis of fatty acids on DNL pathway and incident T2D: FORCE^a^.

Study	Country	Study design	Baseline year(s)	Follow-up years, median	*N* adults (*N* cases)	Age, mean y	Sex, % women	BMI, mean kg/m^2^	Triglycerides, mmol/L	Biomarker fraction
CHS	United States	Cohort	1992	10.6	3,179 (284)	75.1	61.5	26.4	1.57	PL
MESA	United States	Cohort	2000–2002	9.3	2,252 (309)	61.0	53.9	27.6	1.49	PL
IRAS	United States	Cohort	1992–1997	5.3	719 (146)	55.1	55.8	28.4	1.53	Total plasma
FHS	United States	Cohort	2005–2008	5.8	2,209 (98)	64.4	57.2	27.8	1.26	RBC PL
WHIMS	United States	Cohort	1996	11.0	6,510 (502)	70.1	100	28.1	1.56	RBC PL
NHS	United States	Cohort	1990	16.9	1,760 (177)	60.4	100	25.3	N/A	RBC PL, total plasma
HPFS	United States	Cohort	1994	11.1	1,519 (112)	64.1	0	25.8	N/A	RBC PL, total plasma
EPIC-InterAct^b^	8 European countries	Case cohort	1993–1997	12.3	27,296 (12,132)	52.3	62.3	26.0	1.35	PL
AGESR	Iceland	Cohort	2002–2006	5.2	753 (28)	75.5	59.5	27.0	1.14	PL
Three C	France	Cohort	1999–2000	8.0	565 (39)	76.0	64.3	25.0	1.28	RBC PL
AOC	Netherlands	Cohort	2002–2006	2.5	1,741 (201)	68.9	20.4	27.4	1.83	RBC PL, CE
ULSAM	Sweden	Cohort	1970–1973	21.4	2009 (396)	54.4	0	25.2	1.77	Adipose tissue
PIVUS	Sweden	Cohort	2001–2004	10.0	879 (67)	72.5	51.0	26.7	1.24	PL, CE
KIHD	Finland	Cohort	1998–2001	10.3	1,543 (205)	62.7	52.7	27.6	1.23	Total serum
METSIM	Finland	Cohort	2006–2010	5.5	1,302 (71)	57.3	0	26.4	1.35	PL, CE, TG
MCCS	Australia	Case cohort	1990–1994	4.0	6,151 (490)	56.3	53.9	27.0	1.27	PL
CCCC	Taiwan	Cohort	1992–1993	6.0	1,838 (128)	58.7	40.0	23.2	1.29	Total plasma

^a^Baseline characteristics at the time of fatty acid biomarker measurement

^**b**^The EPIC-InterAct Study provided pooled estimates from across 8 European countries: Denmark, France, Germany, Italy, the Netherlands Spain, Sweden, and the United Kingdom.

**Abbreviations:** AGESR: Age, Genes, Environment Susceptibility Study (Reykjavik); AOC, Alpha Omega Cohort; CCCC, Chin-Shan Community Cardiovascular Cohort Study; CE, cholesteryl esters; CHS, Cardiovascular Health Study; FHS, Framingham Heart Study; FORCE, Fatty Acids and Outcomes Research Consortium; HPFS, Health Professionals’ Follow-up Study; KIHD, Kuopio Ischaemic Heart Disease; MCCS, Melbourne Collaborative Cohort Study; MESA, Multi-Ethnic Study of Atherosclerosis; METSIM, Metabolic Syndrome in Men Study; NHS, Nurses’ Health Study; PIVUS, Prospective Investigation of the Vasculature in Uppsala Seniors; PL, phospholipids; RBC, red blood cells; TG, triglycerides; Three C, Three City Study; ULSAM, Uppsala Longitudinal Study of Adult Men; WHIMS, Women’s Health Initiative Memory Study

**Fig 1:**
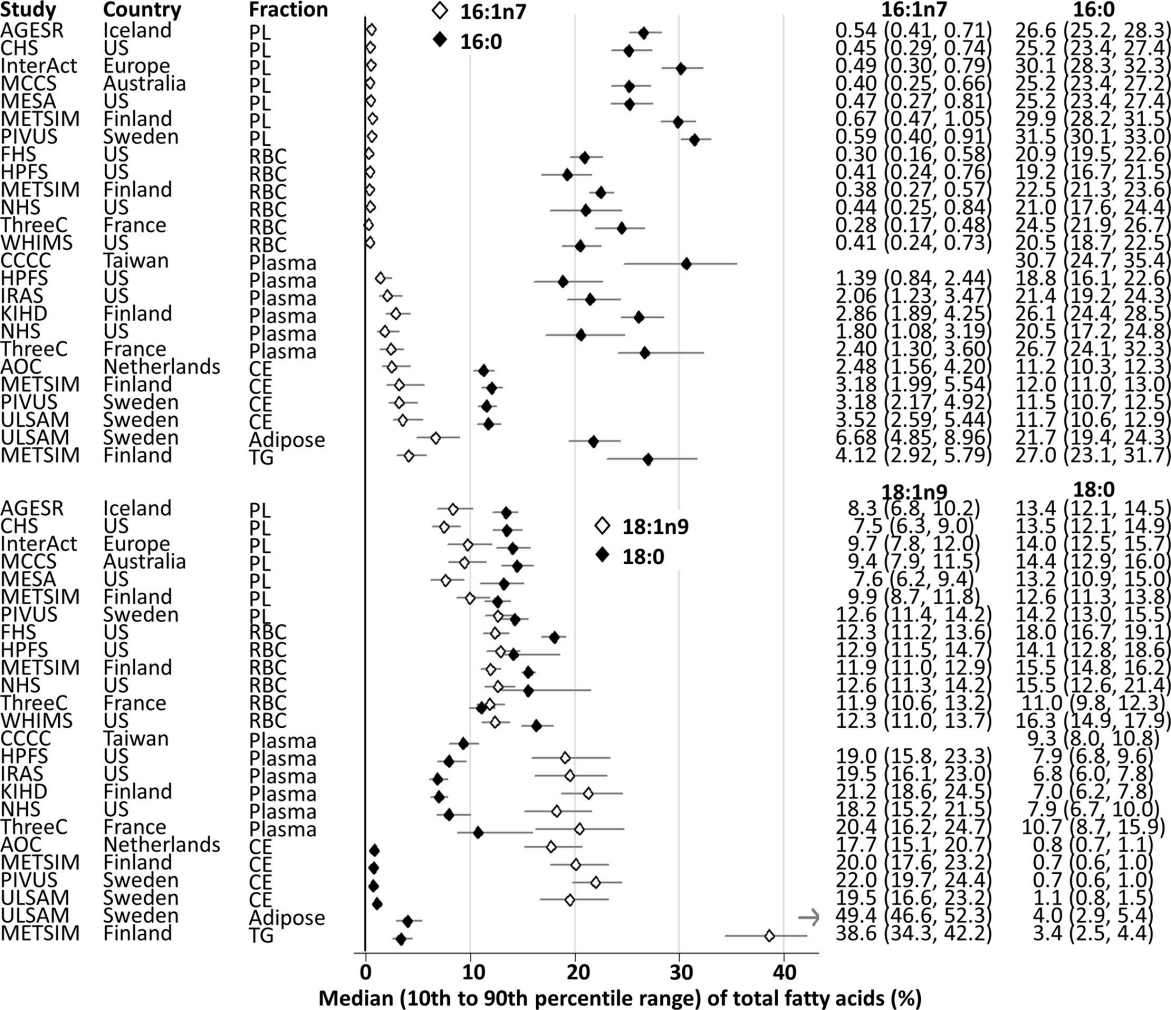
Proportions of fatty acids in the DNL pathway. Plots represent median (diamond) and the range of 10th to 90th percentiles (horizontal bar). See Table 1 for cohort names. CE, cholesteryl ester; DNL, de novo lipogenesis; PL, phospholipid; RBC, red blood cell; US, United States.

The concentrations of the selected fatty acids in the DNL pathway varied by lipid compartment (Fig 1). Concentrations of 16:0 ranged from 15% to 35% of total fatty acids in most lipid compartments except for cholesteryl esters (10% to 13%). Concentrations of 16:1n7 were less than 1.0% when measured in phospholipids (plasma or red blood cell membrane) and 1.0% to 9.0% when measured in the other compartments. In phospholipids, average concentrations of 18:0 were 11.0% to 16.3% and consistently higher than those of 18:1n9. In other lipid compartments, conversely, concentrations of 18:0 were much lower than those of 18:1n9.

Correlations between fatty acids also varied by lipid compartment (S1 Table). For example, in phospholipids, 16:0 positively correlated with 16:1n7 (weighted-average r = 0.47) and 18:1n9 (r = 0.23) but negatively with 18:0 (r = −0.63). By contrast, in cholesteryl ester, triglycerides, and adipose tissue, the correlation between 16:0 and 18:0 was positive (r = 0.39, 0.39, and 0.53, respectively).

### Prospective associations with incident T2D

**Fig 2:**
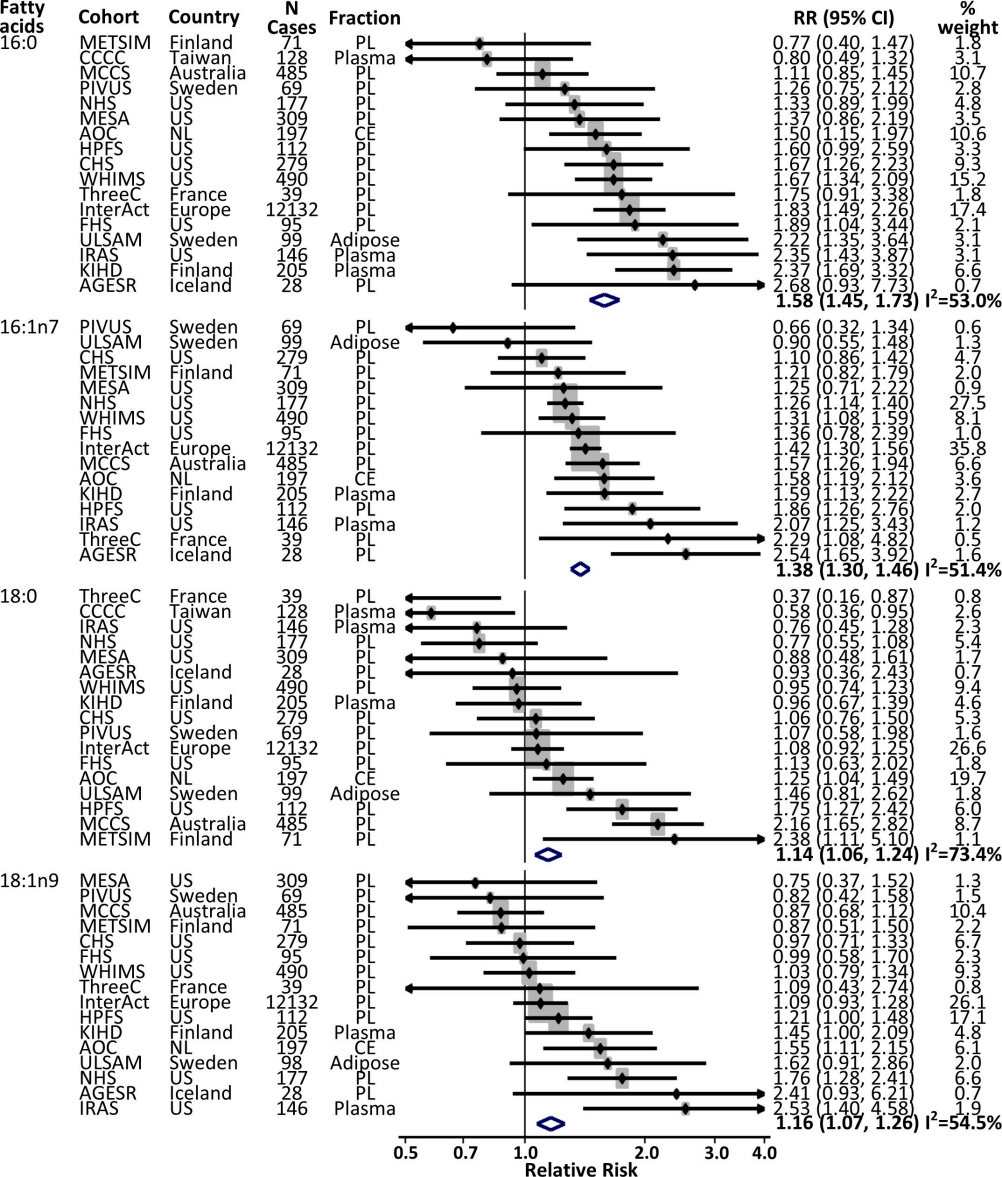
Associations of fatty acids in the DNL pathway with the risk of developing T2D. RRs and 95% CIs are presented in the scale per study-specific range from the midpoints of the first and fifth quintile groups (i.e., 10th to 90th percentiles): dots from individual studies and diamonds as summary estimates meta-analyzed. The sizes of the squares of point estimates represent relative contributions of each cohort to each summary estimate (% weight). Each cohort-specific association was assessed with multivariable-adjusted regression controlling for field site (if appropriate), sex, age, race/ethnicity, socioeconomic characteristics (education, occupation), smoking status, alcohol consumption, physical activity, family history of diabetes, dyslipidaemia, hypertension, menopausal status (women), prevalent coronary heart disease, BMI, and waist circumference. Results remained similar in the other models (S1 Fig and S2 Fig), except for 18:1n9, which showed no significant result in the most adjusted model (*p* = 0.69, S2 Fig). CE, cholesteryl esters; CI, confidence interval; DNL, de novo lipogenesis; PL, phospholipids; RR, relative risk; T2D, type 2 diabetes.

In pooled analyses for each of the 4 fatty acids evaluating a total of 65,225 participants and 15,383 incident T2D cases, significant positive associations were identified, whether before (S1 Fig) or after adjustment (Fig 2) for adiposity measures. For example, RRs (95% CI) per the cohort-specific midpoints of the top and bottom quintiles for 16:0 were 1.63 (1.50–1.76) and 1.53 (1.41–1.66) with and without adjustment for adiposity, respectively (*p* < 0.001 for each). For 16:1n7, 18:0, and 18:1n9, similar or weaker significantly positive associations were observed.

**Fig 3:**
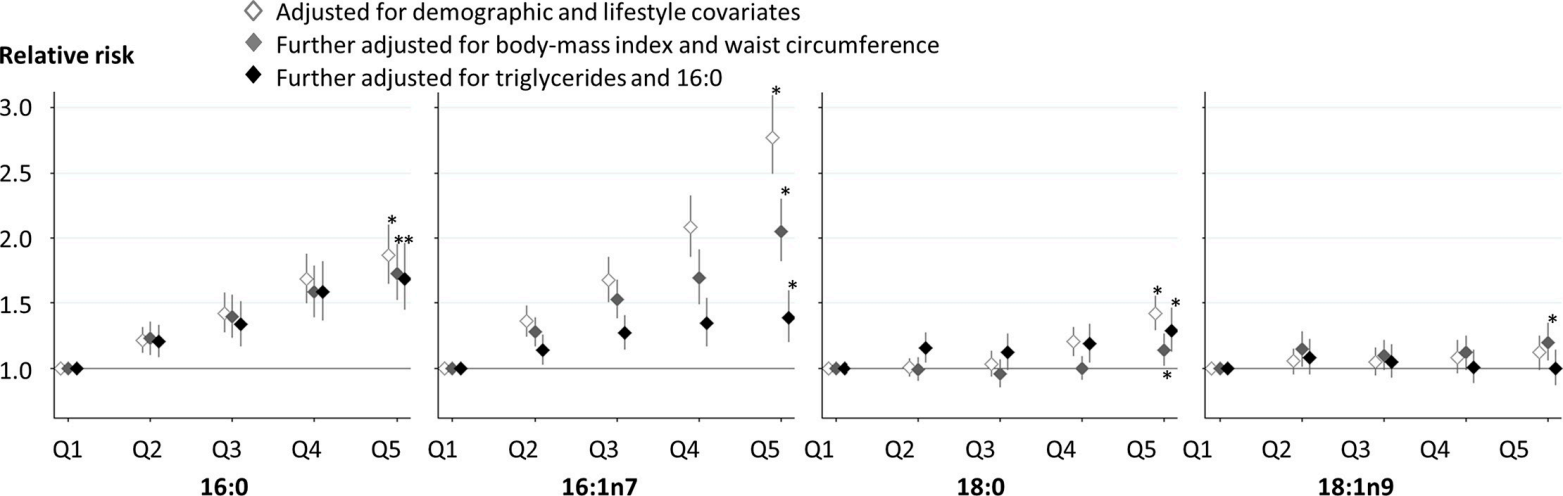
Associations of fatty acids in the DNL pathway with the incidence of T2D. Cohort-specific measures of associations across the quintile groups were pooled with inverse-variance-weighted meta-analysis. In each cohort, 3 different models were fitted: the first, adjusting for study field (if available), sex, age, smoking status, alcohol consumption, socioeconomic status, physical activity, dyslipidaemia, hypertension, and menopausal status (only for women); the second, adjusting for BMI and waist circumference; and the third, adjusting for triglycerides and 16:0 (for 16:1n7, 18:0 and 18:1n9) as the main products of DNL. A trend across quintiles of each fatty acid was tested with meta-analysis of cohort-specific regression coefficients of an ordinal variable of each fatty acid. The association with an asterisk showed *p* < 0.001 except for the second results for 18:0 (*p* = 0.0158) and for 18:1n9 (*p* = 0.0162). BMI, body mass index; DNL, de novo lipogenesis; T2D, type 2 diabetes.

Further adjusting for triglycerides, the association of 16:0 was modestly attenuated, with RR (95% CI) of 1.36 (1.24–1.50; *p* < 0.001; S2 Fig). After adjustment for triglycerides and 16:0, associations of 16:1n7 and 18:0 with T2D incidence were attenuated but still evident with RRs (95% CI) of 1.17 (1.11–1.24; *p* < 0.001) and 1.16 (1.06–1.27; *p* = 0.001), respectively, whereas the association of 18:1n9 with T2D risk was attenuated to the null (RR = 1.03, 95% CI 0.94–1.13, *p* = 0.40). Findings were similar when each fatty acid was evaluated categorically (Fig 3).

Heterogeneity was seen in these pooled analyses, with I^2^ ranging from 52.1% to 73.1% (Fig 2). The between-study heterogeneity was not associated with the global region or lipid fraction (S2 Table, S3–S6 Figs for 16:0, 16:1n7, 18:0, and 18:1n9, respectively). Among post hoc meta-regression analyses, average follow-up years explained heterogeneity of the association of 16:1n7 with T2D risk (S2 Table). I^2^ estimates were 52.1% and 36.8% before and after controlling for follow-up years in meta-regression, respectively. Further stratification of cohorts into those with <10 years and those with ≥10 years of mean follow-up showed RRs (95% CI) of 1.64 (1.43–1.87) and 1.33 (1.25–1.41), respectively. Significant interactions were also not identified by age, sex, race, or BMI, except for sex and 18:1n9 (*p* = 0.002; S7 Fig). In exploratory meta-analysis including cohorts with both sexes and cohorts recruiting only men or women, sex-specific RRs (95% CI) for 18:1n9 were 1.17 (1.05–1.30; *p* = 0.005) for men and 1.10 (0.98–1.24; *p* = 0.10) for women.

## Discussion

In this pooling project using harmonized, de novo individual-participant analyses from 17 prospective cohorts across 12 countries, biomarker concentrations of 16:0, 16:1n7, 18:0, and 18:1n9 were associated with higher risk of T2D. Associations appeared strongest for the 16-carbon saturated and monounsaturated fatty acids, followed by the 18-carbon fatty acids, and were independent of measures of adiposity. The relationships appeared partly confounded or mediated by circulating levels of blood triglycerides, a marker of DNL, although independent associations with T2D remained evident for 16:0, 16:1n7, and 18:0. Statistical heterogeneity between cohorts was largely not explained by age, sex, lipid compartment, or world region. These novel findings across 17 global cohorts suggest that the pathophysiological process of developing T2D is linked to activity of the DNL pathway and/or these circulating fatty acids.

Experimental evidence provides biological plausibility to support these findings; 16:0, the major product of DNL, appears to exert a direct toxic effect on pancreatic cells, activating membrane-bound toll-like receptor 4 and promoting pro-inflammatory responses [26], leading to impaired insulin secretion capacity [4–6]. In cells expressing insulin receptors, 16:0-ceramides attenuate insulin sensitivity by antagonizing the insulin-receptor signaling cascade and impairing endoplasmic reticulum function [27]. DNL also elevate levels of diacylglycerols, which inhibit insulin signaling and impair insulin sensitivity in skeletal muscle [28]. These mechanistic effects support our current findings of a robust positive association between in vivo 16:0 concentrations and incidence of T2D.

16:1n7 positively correlated with 16:0, and mutual adjustment partly but not fully attenuated the association between 16:1n7 and T2D. In rodents, blocking the expression of stearoyl-coenzyme A desaturase 1 gene (SCD), a rate-limiting enzyme for synthesis of 16:1n7 from 16:0, protected against insulin resistance [29]. Our results support the need for future mechanistic investigations of whether 16:1n7 and 16:0 as well as hepatic DNL and SCD activity have overlapping or partly independent roles in the pathogenesis of T2D.

The major modifiable factors that influence circulating levels of 18:0 and 18:1n9 are not well characterized. Lipid compartment-specific analyses of these fatty acids in total plasma/serum versus phospholipids suggested potentially varying associations with T2D, although the availability of cohorts to confirm such heterogeneity was limited. Further work is needed to clarify the determinants, roles, and effects on metabolic risk of 18:0 and 18:1n9 in different lipid fractions, including the potential relevance of DNL versus dietary intakes of these fatty acids.

A number of lifestyle and dietary factors may regulate DNL. Consumption of starch and sugars high in glycemic load are likely to promote DNL by increasing insulin and/or activating the carbohydrate-response pathway in the liver [1,8,9,30–32]. Certain dietary factors, such as coffee and omega-6 polyunsaturated fat, appear to suppress DNL [8,32–35] and are associated with lower incidence of T2D [20,36–38]. Other modifiable factors that may influence DNL include sleeping behavior and meal frequency [39]. Dietary intakes of saturated and monounsaturated fatty acids directly influence 16:0, 16:1n7, 18:0, and 18:1n9 [38,40,41], but it remains unclear whether these effects are similar or even smaller than the influence of endogenous synthesis and metabolism in long-term settings [42–44]. For example, limited evidence from Swedish cohorts suggests the negative or null association of carbohydrate intake with concentrations of DNL-related fatty acids in adipose tissue and phospholipids and highlight a role of saturated fat or alcohol as determining DNL fatty acids [45,46]. Further research should address this uncertainty of dietary carbohydrates and saturated fat in terms of each impact on circulating concentrations of DNL-related fatty acids, genetic activity of *SCD*, and also the accumulation of hepatic fat [35,41]. Further overall mechanistic evidence is crucial to help interpret the current dietary evidence: in contrast to our observed associations based on in vivo circulating biomarkers, dietary monounsaturated fat improves several markers of glucose-insulin homeostasis in randomized feeding trials, but dietary saturated fat has neutral effects compared with dietary carbohydrates [47].

Our analysis has several strengths. We collaboratively pooled new, standardized participant-level analyses across multiple cohorts in various global regions, improving a statistical power from a large number of studies. Our consortium approach should be robust against the potential publication bias. The standardized approaches to defining the populations, exposures, outcomes, and multivariable-adjusted analyses minimized bias and heterogeneity by method.

Our study also has limitations. The diagnosis of T2D could be missed or misclassified in some participants. However, most cohorts operated regular study visits and measurements needed for T2D ascertainment, reducing potential measurement error in the outcome ascertainment, free from bias due to health consciousness leading to T2D screening. Additionally, any outcome misclassification would occur at random across fatty acid measures. As another limitation, we cannot rule out reverse causation that unmeasured diabetes pathophysiology may result in dysregulation of lipolysis, elevate DNL-related fatty acids, and further elevate incident T2D via separate causal pathways. The 16:1n7-T2D association significantly varied by follow-up duration, and this finding could be by chance or regression dilution but may indicate the possibility of reverse causation. This finding only for 16:1n7 may also have reflected a unique role of SCD in the development of T2D. The limitation, nonetheless, indicates the importance of the DNL pathway as a strong noncausal indicator, a causal determinant of T2D risk, or both.

We analyzed fatty acid concentrations and study covariates measured at baseline only. Those measurement errors, temporary variations over time, and unmeasured confounding factors, such as dietary correlates with carbohydrates, fat, and alcohol, could potentially bias our findings in either direction. Of note, the exposure duration represented with a single fatty acid measurement is unclear and likely to vary by laboratory, tissue, fraction type, and cohort settings. A single measure may reflect approximately 3 to 4 weeks of a habitual diet, e.g., according to published kinetic studies on essential fatty acids [24,48]. A 6-month high-carbohydrate diet resulted in higher DNL-related fatty acids concentrations in phospholipids than a 6-month high-fat diet [9]. Isotope-labeling studies related fatty acids to hepatic DNL activity and showed variable responses of specific fatty acids to a diet [49]. However, those observations tend to have been limited in size (e.g., approximately 20 adults in detailed assessments) and represent an acute dietary effect on hepatic DNL (a single meal to a few days), not representing a long-term effect or fatty acids exchanged between circulating lipids and cells [49,50]. Additionally, measures of the DNL-related fatty acids were reproducible over years (correlation coefficients = 0.3–0.7 over 5–18 years) in population-based cohorts [51,52]. Therefore, while temporality of the DNL activity is not clear with our exposure assessment, the DNL-related fatty acids we evaluated are likely to have reflected a “usual” or habitual lifestyle and metabolic status over years.

Statistical between-study heterogeneity was evident but not explained by measured characteristics except for the associations of 16:1n7 with T2D risk systematically varying by follow-up years. A limited number of cohorts investigated certain lipid compartments such as triglycerides and adipose tissue, and laboratory settings were not standardized between cohorts. In addition, some of the observed heterogeneity in this current study could reflect variation in lifestyle factors across the 17 studies. We did not identify significant heterogeneity by race/ethnicity, but the number of participants of non-European descent was relatively limited. The inclusion of only a few cohorts of non-European descent and unknown sources of heterogeneity of the observed associations limit the generalizability of our findings. To better understand the generalizability of our findings and to understand sources of heterogeneity, research in different populations with varying dietary practices is required.

In summary, the current FORCE consortium study including 17 prospective cohorts identified significant associations of higher concentrations of fatty acids related to DNL, especially 16-carbon fatty acids, in relation to incidence of T2D. These findings highlight the potential importance of DNL and these individual fatty acids in the development of T2D, and the need for further investigations on how lifestyle behavioral factors and potential interventions may influence levels of these fatty acids and DNL.

## Supporting information

S1 PRISMA Checklist The PRISMA guideline checklist. PRISMA, Preferred Reporting Items for Systematic Reviews and Meta-Analyses.
(DOCX)

S1 Table Correlations between fatty acids in the DNL pathway. DNL, de novo lipogenesis.
(DOCX)

S2 Table Exploratory analyses of the associations of fatty acids in the DNL pathway with incident T2D. DNL, de novo lipogenesis; T2D, type 2 diabetes.
(DOCX)

S1 Fig Prospective associations of fatty acids in the DNL pathway with the risk of T2D mellitus. DNL, de novo lipogenesis; T2D, type 2 diabetes.
(TIF)

S2 Fig Associations of fatty acids in the DNL pathway with the risk of T2D mellitus after adjustment for 16:0 (for 16:1n7, 18:0, 18:1n9) and triglycerides. DNL, de novo lipogenesis; T2D, type 2 diabetes.
(TIF)

S3 Fig Associations of palmitic acid (16:0), one of the fatty acids in the DNL pathway, with the risk of T2D mellitus: pooled analysis stratified by lipid fraction. DNL, de novo lipogenesis; T2D, type 2 diabetes.
(TIF)

S4 Fig Associations of palmitoleic acid (16:1n7), one of the fatty acids in the DNL pathway, with the risk of T2D mellitus: pooled analysis stratified by lipid fraction. DNL, de novo lipogenesis; T2D, type 2 diabetes.
(TIF)

S5 Fig Associations of stearic acid (18:0), one of the fatty acids in the DNL pathway, with the risk of T2D mellitus: pooled analysis stratified by lipid fraction. DNL, de novo lipogenesis; T2D, type 2 diabetes.
(TIF)

S6 Fig Associations of stearic acid (18:1n9), one of the fatty acids in the DNL pathway, with the risk of T2D mellitus: pooled analysis stratified by lipid fraction. DNL, de novo lipogenesis; T2D, type 2 diabetes.
(TIF)

S7 Fig Forest plots of exp(β) of statistical interaction terms by age, sex, and BMI for an association of each of the fatty acids (16:0, 16:1n7, 18:0, and 18:1n9) related to DNL with incidence of T2D. BMI, body mass index; DNL, de novo lipogenesis; T2D, type 2 diabetes.
(TIF)

S1 Text Characteristics and references of prospective cohorts evaluating associations between fatty acids related to the DNL pathway and the risk of developing T2D. DNL, de novo lipogenesis; T2D, type 2 diabetes.
(DOCX)

S2 Text Study protocol. (DOCX)

## Abbreviations

AOC: Alpha Omega Cohort
BMI: body mass index
CCCC: Chin-Shan Community Cardiovascular Cohort Study
CHS: Cardiovascular Health Study
CI: confidence interval
DNL: de novo lipogenesis
FORCE: Fatty Acids and Outcomes Research Consortium
MCCS: Melbourne Collaborative Cohort Study
MESA: Multi-Ehtnic Study of Atheroscerlosis
PRISMA: Preferred Reporting Items for Systematic Reviews and Meta-Analyses
RR: relative risk
SCD: stearoyl-coenzyme A desaturase 1 gene
T2D: type 2 diabetes
WHIMS: Women’s Health Initiative Memory Study
