# Supplementary material for: Fatty acids in the de novo lipogenesis pathway and incidence of type 2 diabetes: A pooled analysis of prospective cohort studies
Source: PLoS Med. 2020 Jun 12;17(6):e1003102. doi: 10.1371/journal.pmed.1003102 (PMC7292352; doi:10.1371/journal.pmed.1003102)
Supplement: S1 Table — DNL, de novo lipogenesis. (DOCX) [file pmed.1003102.s002.docx]

| ****S1 Table. Correlations between fatty acids in the de novo lipogenesis pathway**** | | | | | | | |
| --- | --- | --- | --- | --- | --- | --- | --- |
| **Fraction and study^1^** | **N** | **Pearson correlation coefficients between fatty acid variables^3^** | | | | | |
|  |  | **16:0, 16:1n7** | **16:0,  18:0** | **16:0, 18:1n9** | **16:1n7, 18:0** | **16:1n7, 18:1n9** | **18:0, 18:1n9** |
| **Phospholipids** |  |  |  |  |  |  |  |
| AGESR | 753 | 0.44 | -0.72 | 0.36 | -0.25 | 0.63 | -0.27 |
| CHS | 3179 | 0.63 | -0.53 | 0.34 | -0.31 | 0.61 | -0.26 |
| InterAct | 16164 | 0.42 | -0.70 | 0.20 | -0.32 | 0.55 | -0.26 |
| MCCS | 5712 | 0.49 | -0.59 | 0.24 | -0.24 | 0.49 | -0.19 |
| MESA | 2252 | 0.54 | -0.27 | 0.24 | -0.22 | 0.55 | -0.07 |
| METSIM | 1302 | 0.57 | -0.66 | 0.37 | -0.32 | 0.67 | -0.26 |
| PIVUS | 879 | 0.23 | -0.41 | 0.16 | -0.22 | 0.53 | -0.32 |
| **Red blood cells** |  |  |  |  |  |  |  |
| FHS | 2209 | 0.66 | -0.50 | -0.09 | -0.56 | 0.25 | -0.27 |
| HPFS | 1519 | 0.53 | 0.26 | 0.14 | -0.04 | 0.42 | -0.32 |
| METSIM | 1302 | 0.45 | -0.18 | 0.24 | -0.17 | 0.3 | -0.12 |
| NHS | 1760 | 0.65 | -0.12 | 0.21 | -0.37 | 0.5 | -0.37 |
| ThreeC | 565 | 0.41 | -0.34 | 0.39 | -0.24 | 0.34 | -0.22 |
| WHIMS | 6510 | 0.51 | -0.05 | 0.22 | -0.34 | 0.36 | -0.19 |
| **Plasma/serum** |  |  |  |  |  |  |  |
| CCCC | 1838 |  | 0.11 |  |  |  |  |
| HPFS | 1519 | 0.76 | -0.21 | 0.36 | 0.76 | 0.46 | 0.36 |
| IRAS | 719 | 0.73 | -0.28 | 0.42 | -0.33 | 0.5 | -0.31 |
| KIHD | 1543 | 0.63 | -0.28 | 0.39 | -0.28 | 0.42 | -0.41 |
| NHS | 1760 | 0.65 | -0.12 | 0.21 | -0.37 | 0.5 | -0.37 |
| ThreeC | 565 | -0.12 | 0.60 | -0.51 | -0.32 | 0.43 | -0.61 |
| **Cholesteryl esters** |  |  |  |  |  |  |  |
| AOC | 760 | 0.34 | 0.34 | 0.43 | -0.10 | 0.63 | 0.14 |
| METSIM | 1302 | 0.38 | 0.33 | 0.32 | -0.03 | 0.69 | 0.16 |
| PIVUS | 879 | 0.22 | 0.35 | 0.29 | -0.10 | 0.59 | 0.14 |
| ULSAM | 2009 | 0.39 | 0.47 | 0.58 | -0.08 | 0.63 | 0.24 |
| **Triglycerides** |  |  |  |  |  |  |  |
| METSIM | 1302 | 0.49 | 0.53 | -0.28 | -0.02 | -0.14 | -0.17 |
| **Adipose tissue** |  |  |  |  |  |  |  |
| ULSAM | 2009 | -0.29 | 0.49 | -0.36 | -0.67 | 0.44 | -0.30 |
| **Summary by fraction**^2^ |  |  |  |  |  |  |  |
| Phospholipids |  | 0.47 | -0.63 | 0.23 | -0.29 | 0.55 | -0.23 |
| RBC |  | 0.55 | -0.13 | 0.17 | -0.33 | 0.36 | -0.24 |
| Plasma/serum |  | 0.64 | -0.08 | 0.25 | -0.01 | 0.46 | -0.23 |
| Cholesteryl esters |  | 0.35 | 0.39 | 0.45 | -0.07 | 0.64 | 0.19 |
| Triglycerides |  | 0.49 | 0.53 | -0.28 | -0.02 | -0.14 | -0.17 |
| Adipose tissue |  | -0.29 | 0.49 | -0.36 | -0.67 | 0.44 | -0.30 |

^1^ AGES-Reykjavik, Age, Genes, Environment Susceptibility Study (Reykjavik); AOC, Alpha Omega Cohort; CCCC, Chin-Shan Community Cardiovascular Cohort Study; CHS, Cardiovascular Health Study; FHS, Framingham Heart Study; HPFS, Health Professionals’ Follow-up Study; MESA, Multi-Ethnic Study of Atherosclerosis; METSIM, Metabolic Syndrome in Men Study; NHS, Nurses’ Health Study; PIVUS, Prospective Investigation of the Vasculature in Uppsala Seniors; Three C, Three City Study; ULSAM, Uppsala Longitudinal Study of Adult Men; WHIMS, Women’s Health Initiative Memory Study. Melbourne Collaborative Cohort Study and cholesteryl esters components of PIVUS measured a single dairy fatty acid, and thus correlation coefficients were not available. RBC, red blood cells.

^2^ Each average of correlation coefficients was calculated through Fisher Z-transformation.
