# Supplementary material for: Fatty acids in the de novo lipogenesis pathway and incidence of type 2 diabetes: A pooled analysis of prospective cohort studies
Source: PLoS Med. 2020 Jun 12;17(6):e1003102. doi: 10.1371/journal.pmed.1003102 (PMC7292352; doi:10.1371/journal.pmed.1003102)
Supplement: S2 Table — DNL, de novo lipogenesis; T2D, type 2 diabetes. (DOCX) [file pmed.1003102.s003.docx]

**S2 Table. Exploratory analyses of the associations of fatty acids in the de novo lipogenesis pathway with incident type 2 diabetes.**

|  | **N studies^1^** | **Palmitic acid**  **(16:0)** | | **Palmitoleic acid**  **(16:1n-7)** | | **Stearic acid**  **(18:0)** | | **Oleic acid**  **(18:1n-9)** | |
| --- | --- | --- | --- | --- | --- | --- | --- | --- | --- |
|  |  | **RR (95% CI)** | ***p*^2^** | **RR (95% CI)** | ***p*^2^** | **RR (95% CI)** | ***p*^2^** | **RR (95% CI)** | ***p*^2^** |
| Main pooled estimates | 17 | 1.53 (1.41, 1.66) |  | 1.40 (1.33, 1.48) |  | 1.14 (1.05, 1.22) |  | 1.16 (1.07, 1.25) |  |
| Random-effects estimates | 17 | 1.56 (1.36, 1.80) |  | 1.41 (1.27, 1.56) |  | 1.09 (0.91, 1.29) |  | 1.19 (1.04, 1.37) |  |
| Regions |  |  |  |  |  |  |  |  |  |
| United States | 7 | 1.65 (1.44, 1.89) |  | 1.29 (1.19, 1.40) |  | 1.04 (0.90, 1.19) |  | 1.20 (1.07, 1.36) |  |
| Europe/Australia | 9 | 1.60 (1.42, 1.80) |  | 1.45 (1.35, 1.56) |  | 1.23 (1.12, 1.36) |  | 1.12 (1.01, 1.25) |  |
| Asia | 1 | 0.80 (0.49, 1.32) | 0.12 | Not available | 0.12 | 0.58 (0.36, 0.95) | 0.24 | Not available | 0.71 |
| Lipid fractions^3^ |  |  |  |  |  |  |  |  |  |
| Phospholipids | 13 | 1.53 (1.39, 1.70) |  | 1.37 (1.29, 1.45) |  | 1.17 (1.06, 1.28) |  | 1.09 (1.00, 1.20) |  |
| Total plasma | 6 | 1.84 (1.57, 2.15) |  | 1.76 (1.48, 2.09) |  | 0.76 (0.63, 0.91)^4^ |  | 1.73 (1.41, 2.11)^4^ |  |
| Cholesteryl esters | 4 | 1.39 (1.18, 1.64) |  | 1.52 (1.31, 1.76) |  | 1.21 (1.06, 1.38) |  | 1.25 (1.04, 1.51) |  |
| Adipose tissue | 1 | 2.22 (1.35, 3.64) |  | 0.90 (0.55, 1.48) |  | 1.46 (0.81, 2.62) |  | 1.62 (0.91, 2.85) |  |
| Triglycerides | 1 | 1.44 (0.80, 2.61) | >0.1^4^ | 1.05 (0.62, 1.75) | >0.1^4^ | 1.51 (0.93, 2.45) | >0.1^4^ | 0.85 (0.49, 1.48) | >0.1^4^ |
| Estimates after converting odds ratios to risk ratios^5^ | 17 | 1.58 (1.45, 1.73) |  | 1.38 (1.31, 1.45) |  | 1.13 (1.02, 1.21) |  | 1.16 (1.06, 1.26) |  |
| Cohorts estimating hazard ratios | 15 | 1.46 (1.34, 1.56) |  | 1.37 (1.30, 1.45) |  | 1.12 (1.03, 1.22) |  | 1.12 (1.03, 1.23) |  |
| Cohorts estimating odds ratios^6^ | 2 | 1.87 (1.47, 2.37) | 0.34 | 1.56 (1.24, 2.00) | 0.52 | 1.48 (1.14, 1.92) | 0.93 | 1.50 (1.17, 1.92) | 0.26 |
| Follow-up years^7^ |  |  |  |  |  |  |  |  |  |
| <10 years | 9 | 1.34 (1.15, 1.56) |  | 1.64 (1.43, 1.87) |  | 1.28 (1.12, 1.45) |  | 1.15 (0.97, 1.35) |  |
| ≥10 years | 8 | 1.73 (1.55, 1.92) | 0.18 | 1.33 (1.25, 1.41) | 0.03 | 1.07 (0.97, 1.18) | 0.70 | 1.16 (1.06, 1.28) | 0.50 |
| Meta-regression estimates^8^ |  |  |  |  |  |  |  |  |  |
| Follow-up years, per 5 years | 17 | 1.12 (0.96, 1.30) | 0.18 | 0.91 (0.84, 0.98) | 0.03 | 0.96 (0.77, 1.19) | 0.70 | 1.06 (0.90, 1.26) | 0.50 |
| Proportions of dyslipidemia | 17 | 1.16 (0.62, 2.16) | 0.64 | 1.20 (0.70, 2.07) | 0.51 | 1.05 (0.45, 2.45) | 0.90 | 1.41 (0.75, 2.67) | 0.31 |
| Proportions of hypertension | 17 | 1.31 (0.69, 2.49) | 0.42 | 1.54 (0.95, 2.49) | 0.10 | 0.64 (0.27, 1.47) | 0.31 | 1.50 (0.78, 2.87) | 0.24 |
| Mean triglycerides, per 0.5 mM | 17 | 1.15 (0.74, 1.78) | 0.55 | 0.81 (0.58, 1.14) | 0.26 | 1.12 (0.66, 1.91) | 0.69 | 1.27 (0.85, 1.91) | 0.27 |
| Proportions of fasting blood^9^ | 17 | 1.03 (0.65, 1.62) | 0.91 | 0.89 (0.61, 1.29) | 0.54 | 0.72 (0.40, 1.29) | 0.29 | 0.90 (0.56, 1.45) | 0.66 |

^1^ The numbers varied by fatty acid variable, and the total number of cohorts used is presented. The total number of cohorts was seventeen. RR and CI stand for relative risk and confidence interval.

^2^ P values for heterogeneity were obtained using meta-regression.
^3^ In the analysis stratifying lipid fractions, cohorts measuring fatty acids in multiple lipid fractions (NHS, HPFS, PIVUS, ULSAM, and METSIM) contributed to multiple strata. In the meta-regression to assess heterogeneity, double counting was avoided by excluding estimates from single cohorts. For example, to test the difference between triglyceride fatty acids (METSIM only) and phospholipid fatty acids, METSIM’s measures of phospholipid fatty acids were excluded. Meta-analyses double-counting estimates from single cohorts, being over-powered, also produced similar findings, with exception for the 18:1 n-9 (see footnote 4).

^4^ The positive association of total plasma/serum 18:1 n-9 was stronger than that of phospholipids and of triglycerides (p=0.0003 and 0.04, respectively). After adjustment for triglycerides and 16:0, associations in the five strata became not significant.
^5^ Two cohorts (Alpha Omega Cohort and Melbourne Collaborative Cohort Study) provided odds ratios (OR), violating a rare-disease assumption or unlikely producing odds ratios not approximating risk ratios. Inclusion of ORs as they were could be inappropriate. To correct the potential bias of over-estimation of risk ratios and subsequent pooled results, we (i) calculated degree of the bias by converting ORs to risk ratios in categorical analyses (Zhang et al., JAMA, 1998;280(19):1690-1691), (2) conducted dose-response linear-trend analysis (Greenland et al., Am J Epidemiol, 1992; 135(11):1301-9), and (3) calculated the relative difference between betas before and after the conversion (a ratio of two betas for a linear trend). Then, (4) we applied the measure of bias as a correction factor to the OR from a continuous approach (used for Fig2), correcting ORs from AOC and MCCS, separately, where standard errors were further recalculated by keeping z-score (beta / standard error) consistent before and after the correction; and (5) re-conducted meta-analysis as in the primary analysis.

^6^ The corresponding meta-regression and within-stratum meta-analysis were conducted after converting ORs to risk ratios as written above^5^.

^7^ According to the post-hoc meta-regression results, meta-analysis stratified by follow-up year was performed explanatorily. P-values represent ones from meta-regression treating follow-up years as a cohort-specific continuous variable.
^8^ Except for proportions of dyslipidemia, analyses were post-hoc. Parameter estimates indicate the ratio of two RRs of two different conditions (e.g. RR in a hypothetical population with dyslipidemia / RR in a hypothetical population without dyslipidemia). For follow-up years and average triglycerides, RR indicates a relative difference in RR per difference by 5 years and 0.5 mmol/L, respectively. Table 1 of the main text lists the distribution of each.

^9^ Most of the cohorts collected fasting bloods except for five: Alpha-Omega Cohort, 0%; Melbourne Collaborative Cohort Study, % of samples of fasting blood=67.3%; EPIC-InterAct, 22.7%; Health Professionals’ Follow-up Study, 59.1%; and Nurses’ Health Study, 71.9%.
