# Supplementary figures and images for: Fatty acids in the de novo lipogenesis pathway and incidence of type 2 diabetes: A pooled analysis of prospective cohort studies
Source: PLoS Med. 2020 Jun 12;17(6):e1003102. doi: 10.1371/journal.pmed.1003102 (PMC7292352; doi:10.1371/journal.pmed.1003102)

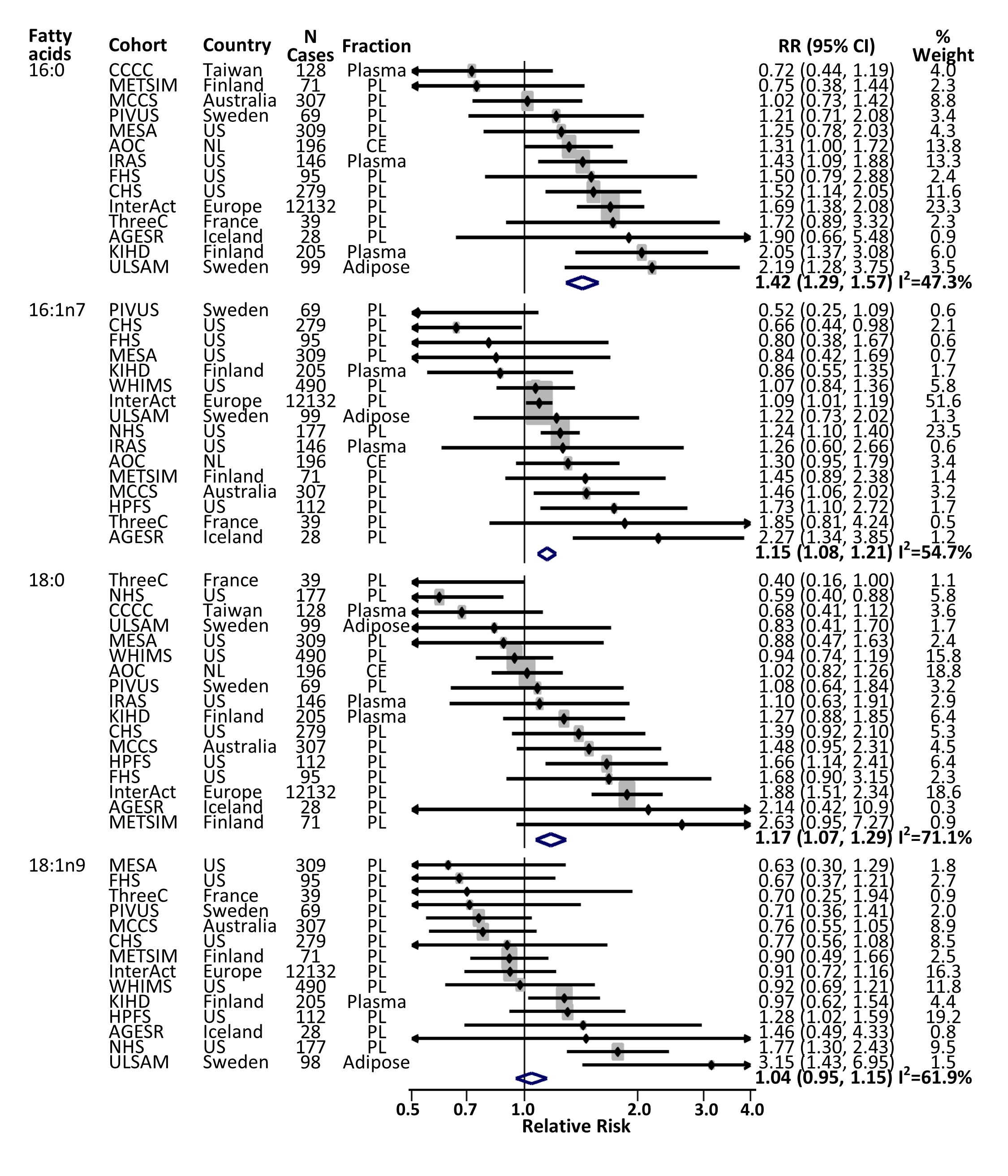

Supplement: S1 Fig — DNL, de novo lipogenesis; T2D, type 2 diabetes. (TIF) [file pmed.1003102.s004.TIF]

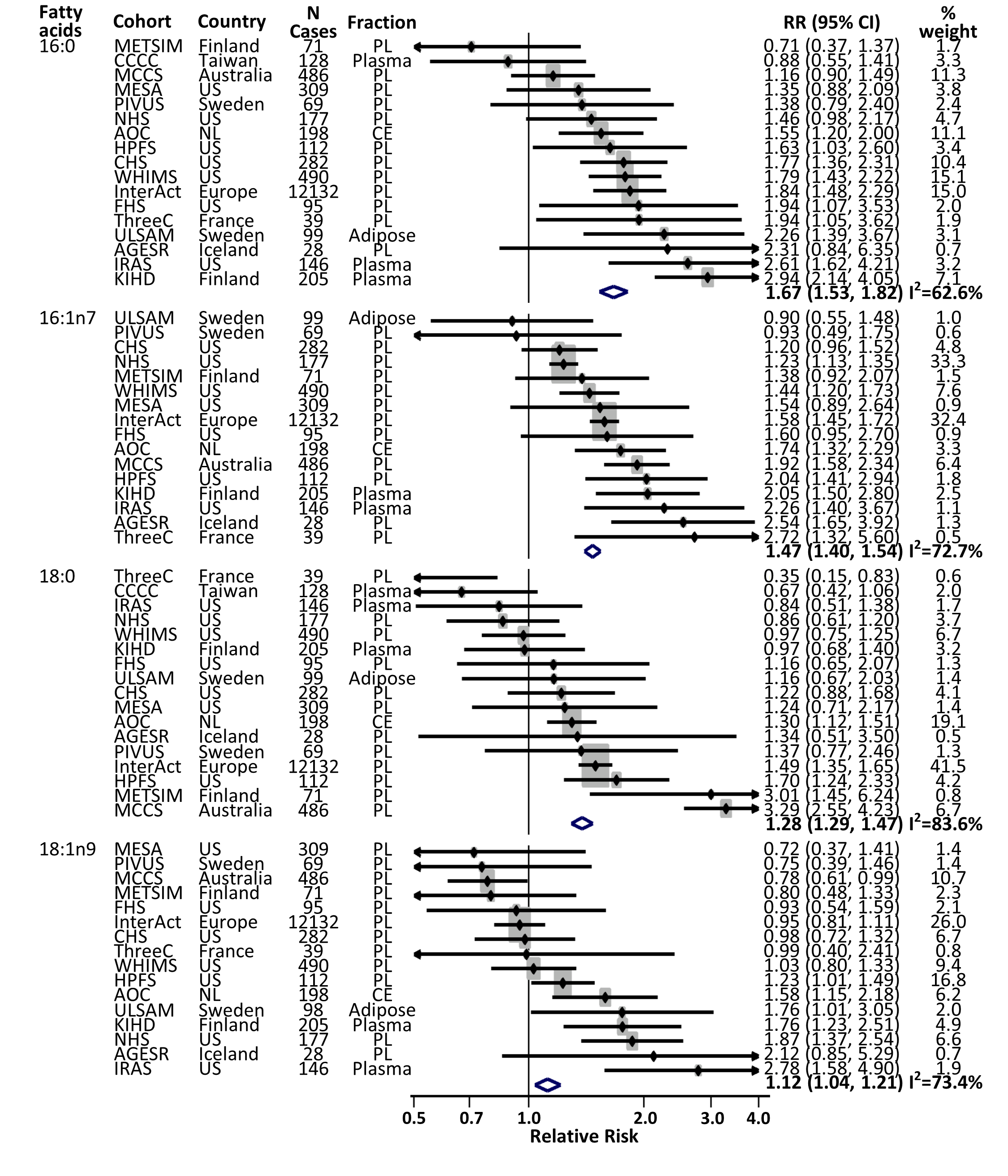

Supplement: S2 Fig — DNL, de novo lipogenesis; T2D, type 2 diabetes. (TIF) [file pmed.1003102.s005.TIF]

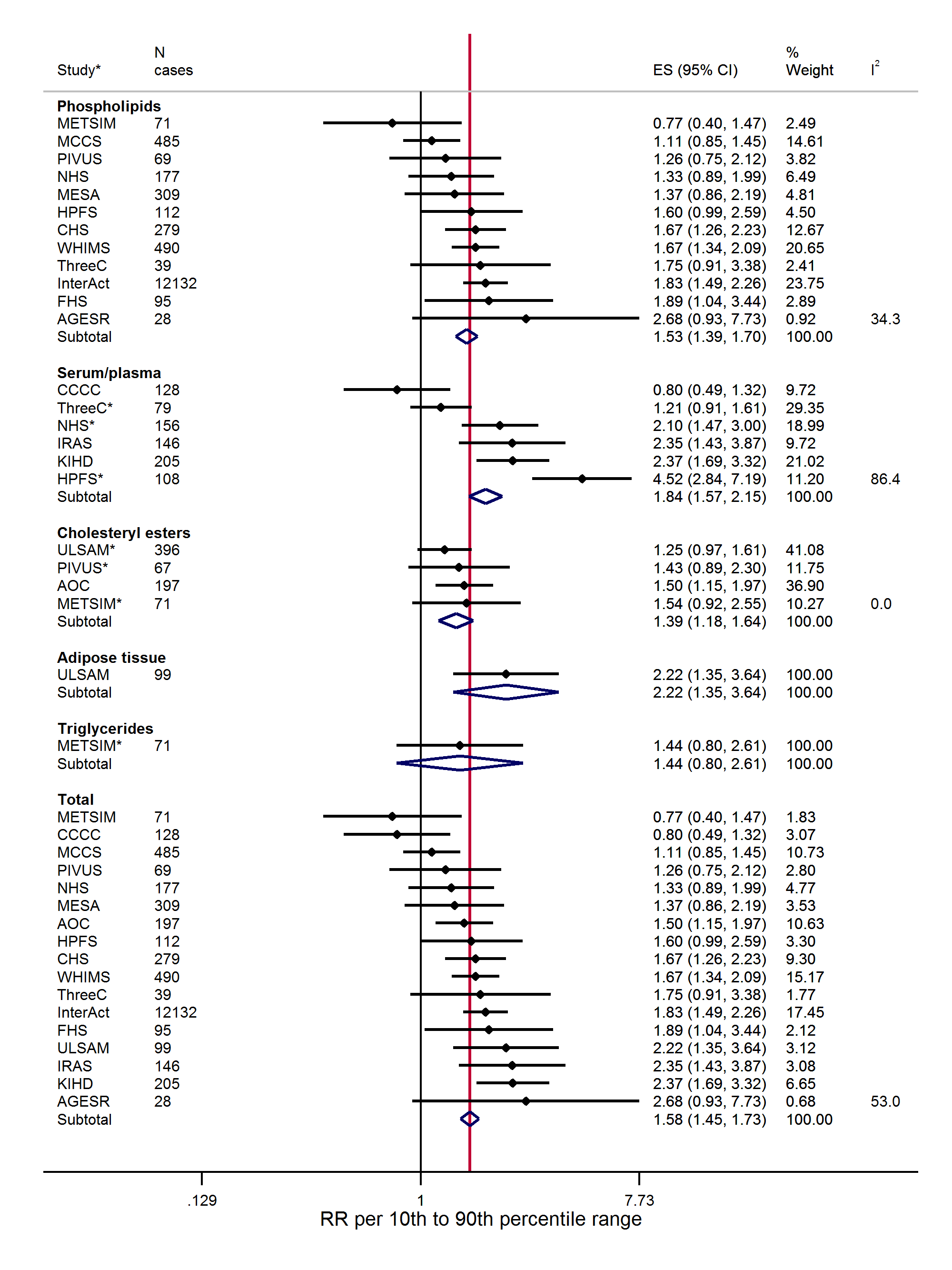

Supplement: S3 Fig — DNL, de novo lipogenesis; T2D, type 2 diabetes. (TIF) [file pmed.1003102.s006.tif]

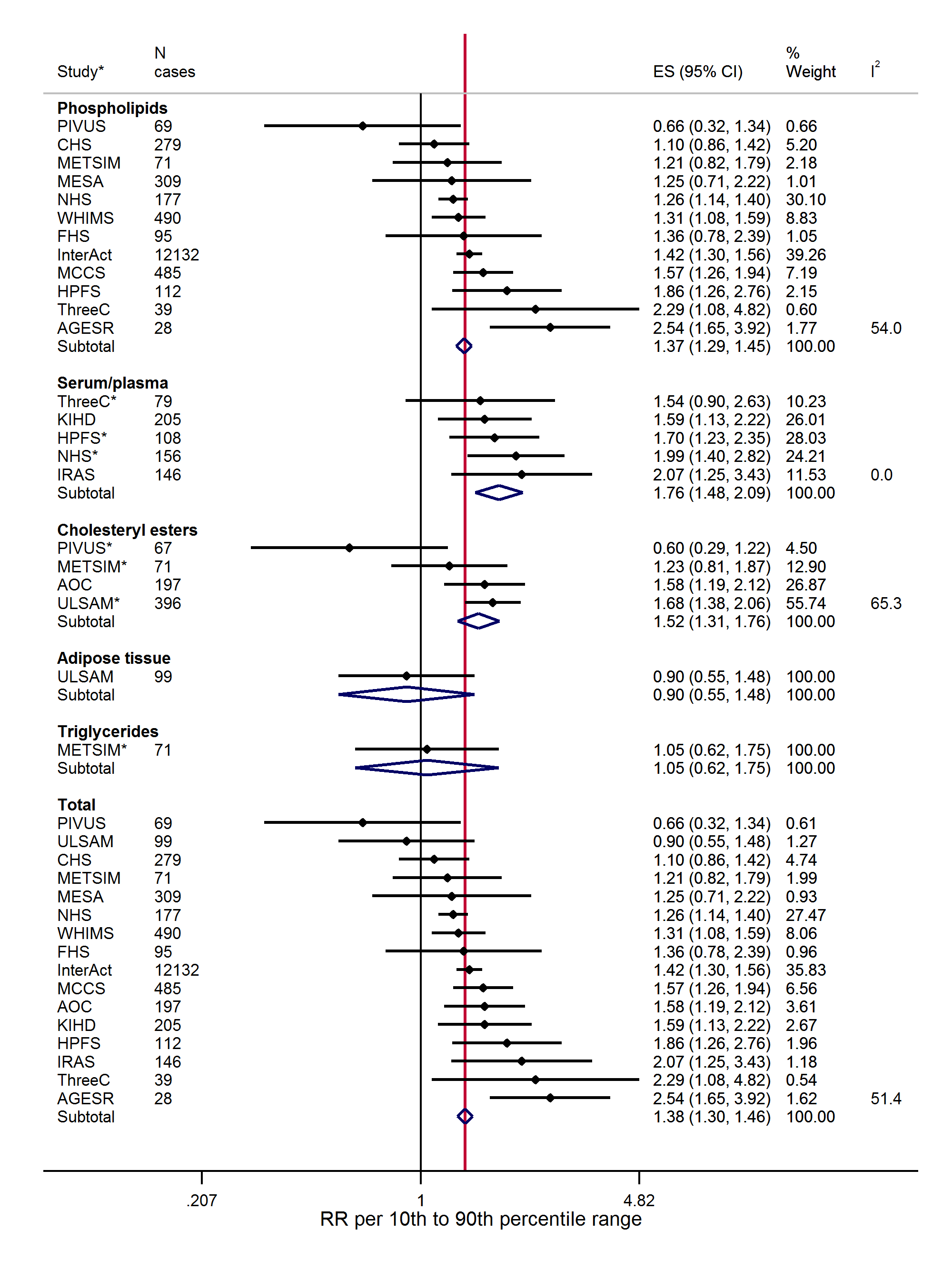

Supplement: S4 Fig — DNL, de novo lipogenesis; T2D, type 2 diabetes. (TIF) [file pmed.1003102.s007.tif]

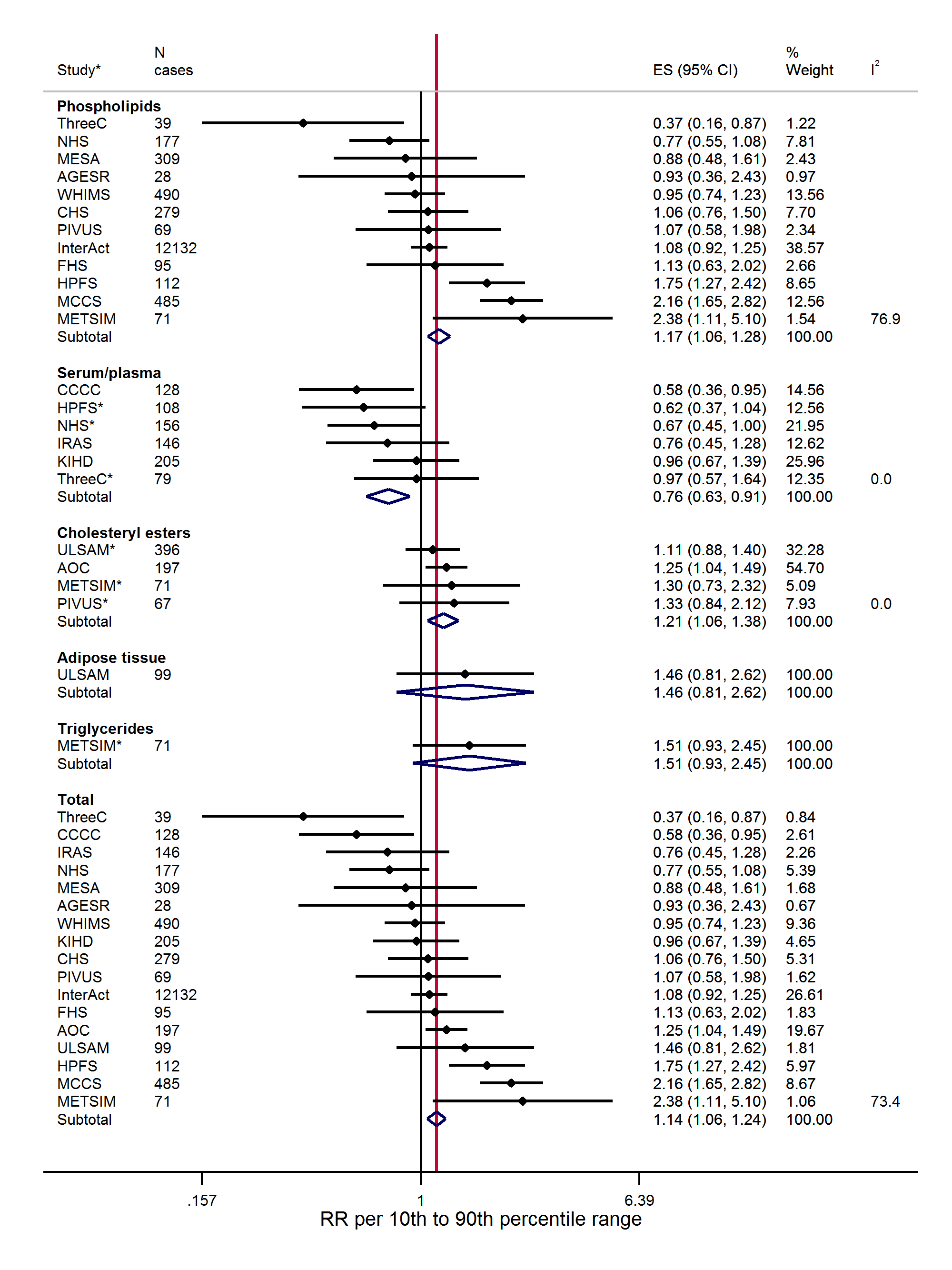

Supplement: S5 Fig — DNL, de novo lipogenesis; T2D, type 2 diabetes. (TIF) [file pmed.1003102.s008.tif]

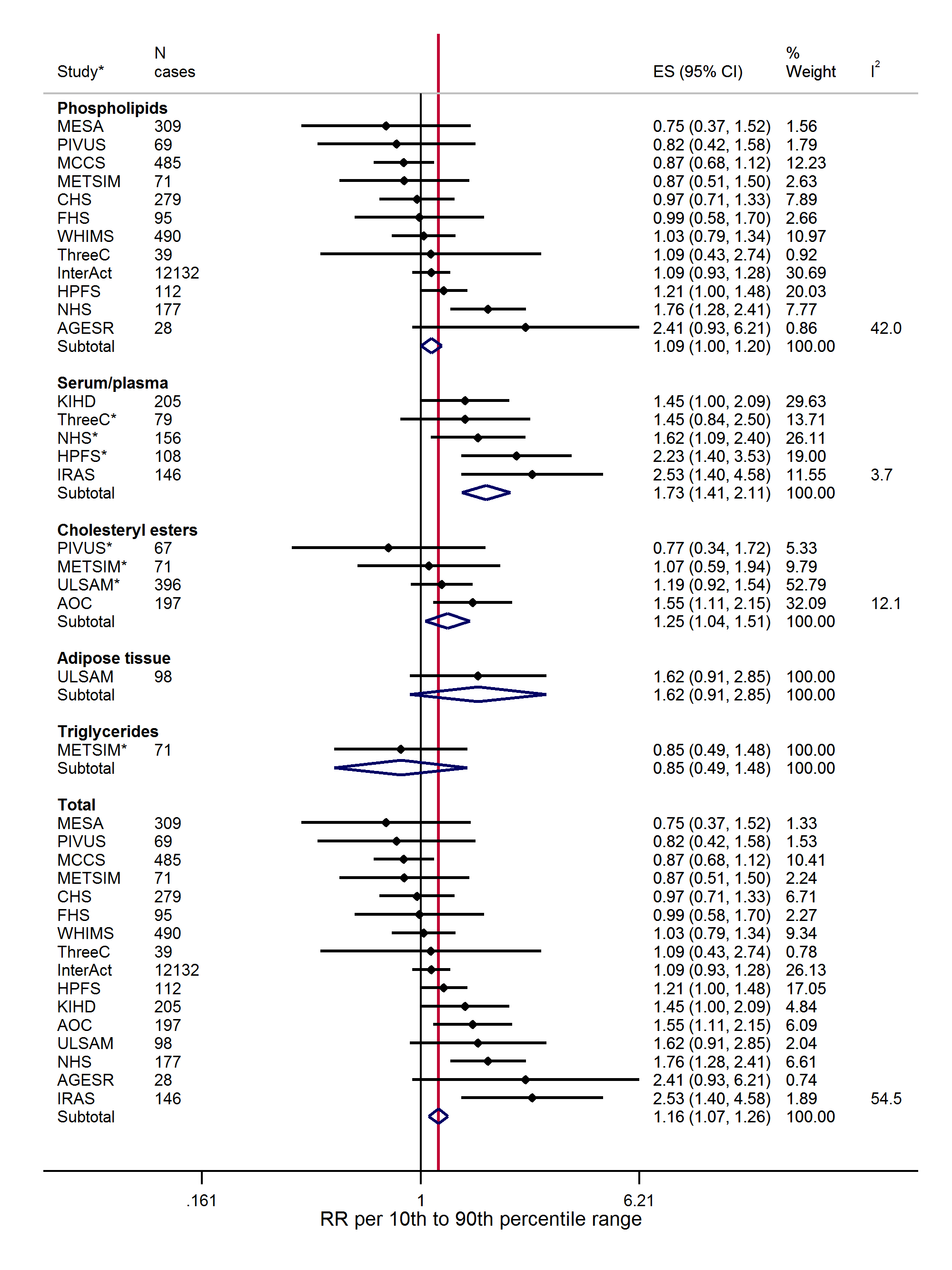

Supplement: S6 Fig — DNL, de novo lipogenesis; T2D, type 2 diabetes. (TIF) [file pmed.1003102.s009.tif]

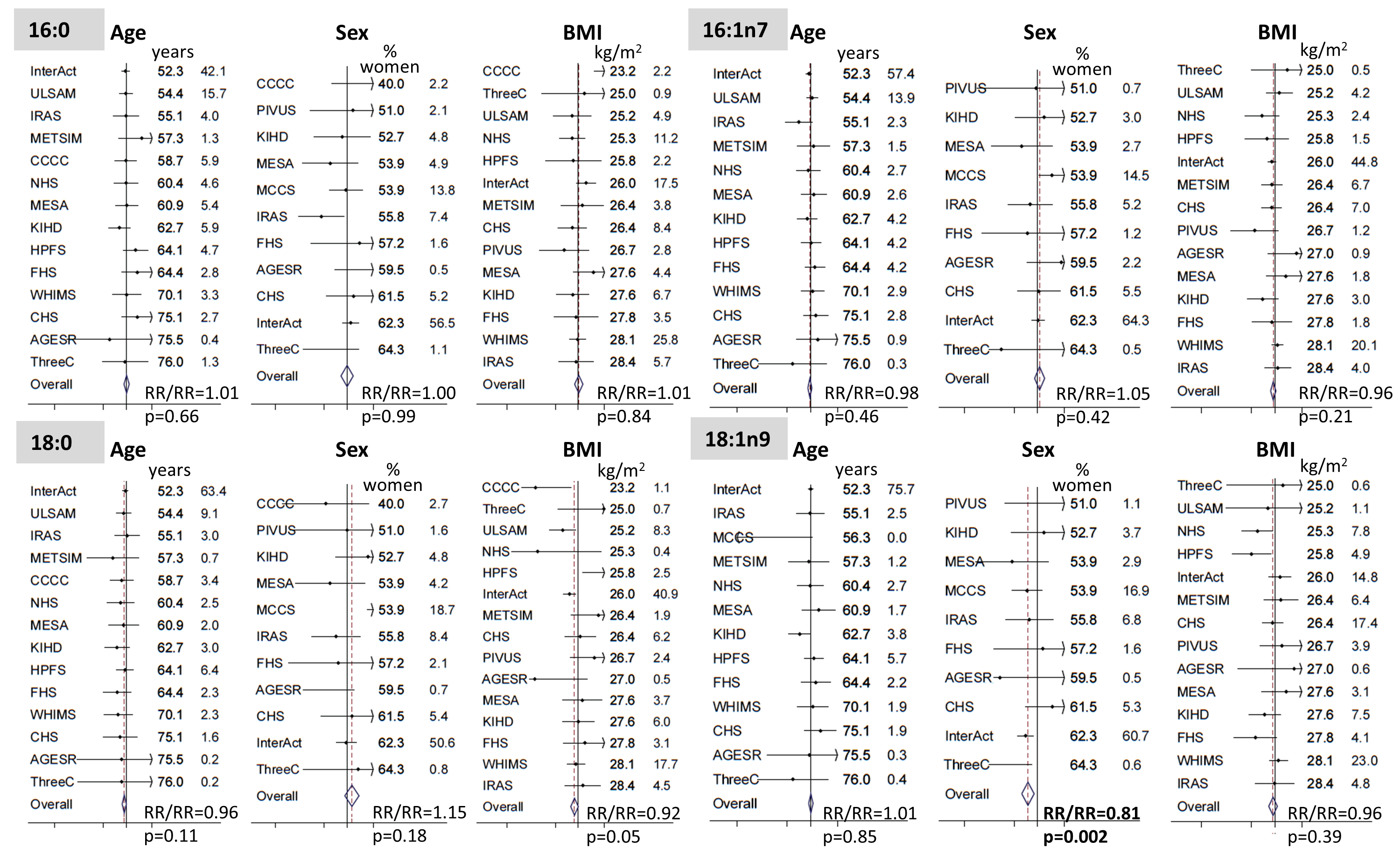

Supplement: S7 Fig — BMI, body mass index; DNL, de novo lipogenesis; T2D, type 2 diabetes. (TIF) [file pmed.1003102.s010.TIF]
