# Supplementary material for: Fatty acids in the de novo lipogenesis pathway and incidence of type 2 diabetes: A pooled analysis of prospective cohort studies
Source: PLoS Med. 2020 Jun 12;17(6):e1003102. doi: 10.1371/journal.pmed.1003102 (PMC7292352; doi:10.1371/journal.pmed.1003102)
Supplement: S2 Text — (DOCX) [file pmed.1003102.s012.docx]

**S2 Text. Study Protocol

Title:** Circulating Fatty Acids in the De Novo Lipogenesis Pathway and Incident Diabetes: a Meta-Analysis of Prospective Cohort Studies

Lead authors: Nita G Forouhi, Fumiaki Imamura, Amanda Fretts, Rozenn N Lemaitre, Dariush Mozaffarian

Correspondence:

Fumiaki Imamura, PhD MS

University of Cambridge, MRC Epidemiology Unit

Cambridge Biomedical Campus, Cambridge, CB2 0QQ, UK

fumiaki.imamura@mrc-epid.cam.ac.uk

Background:

De novo lipogenesis (DNL) is a metabolic pathway for synthesis of triglycerides and other lipids.^1,2^ Individual fatty acids in the DNL pathway include palmitic acid (16:0), *cis* palmitoleic acid (c16:1n-7), stearic acid (18:0), and oleic acid (c18:1n-9). Tissue levels of these fatty acids have been known to be higher among adults with diabetes compared to healthy adults and be related to insulin resistance.^3^ Thus, these DNL-derived fatty acids may be risk factors for incident type 2 diabetes, one of the main causes of morbidity and mortality across the world.^4^

A few biological experiments have supported causal detrimental effects of 16:0 on inflammatory response and pancreatic function, whereas protective effects of c18:1n9 on pancreatic function have been suggested.^5–8^ In addition, greater activity of DNL is known to reflect higher consumption of carbohydrates and alcohol, while these DNL-derived fatty acids are present in typical sources of dietary fats.^9–12^ Although no clear quantitative evidence is available about relative contributions of DNL and a habitual diet to the DNL-derived fatty acids in human tissues, research relating DNL-derived fatty acids to incident T2D may provide implications for aetiological knowledge and help identification of modifiable lifestyle risk factors.

Several studies examined circulating DNL-derived fatty acids for their associations with incident T2D.^13–17^ Studies in Sweden and Australia showed null associations between each of 16:0, c16:1n7, 18:0 and c18:1n9 and incident T2D.^13,16^ A German study showed a positive association of c16:1n7 only.^15^ A US study related fatty acid levels of phsopholipids and cholesteryl esters separately to incident T2D and reported complex results: positive associations with T2D was found for 16:0 of both lipid fractions, phospholipid 18:0, and cholesteryl-ester c16:1n7; and an inverse association was found for phospholipid c18:1n9.^17^ A British study evaluated fatty acids of erythrocytes and phospholipids separately and reported positive associations of 16:0 of both fractions, a positive association of erythrocyte c16:1n7, and an inverse association of phospholipid 18:0.^14^ Thus, past evidence is inconclusive. The inconsistency partly depends on lipid fractions and on a limited size of each study (N cases<800). Differences in demographics and study designs might also contribute to the heterogeneity of results.

To examine the association with a large sample size and examine sources of the heterogeneity, we propose to test the hypothesis that circulating levels of DNL-derived fatty acids are associated with incident T2D as the project of FORCE.

**PROJECT AIM:**

To examine the association of the following fatty acids with incident T2D:

(a) 16:0

(b) c16:1n7

(c) 18:0

(d) c18:1n9

**METHODS:**

Sample:

- Adults (aged 18+ years) from the CHARGE Fatty Acid Phenotype working group with measures of circulating 16:0, c16:1n7, 18:0, and c18:1n9

Both cohort studies and case-control studies with available data on any of circulating 16:0, c16:1n7, 18:0, and c18:1n9 and incident diabetes data are eligible to participate in the project.

Exclusions:

- Participants <18 years of age
- Prevalent diabetes
- No data on any of circulating 16:0, c16:1n7, 18:0, and c18:1n9

Dependent Variable:

- incident diabetes (as defined by the cohort)

Independent variables:

1. biomarker 16:0;
2. biomarker c16:1n7;
3. biomarker 18:0;
4. biomarker c18:1n9;

If more than one fatty acid biomarker is available (e.g., the study has data available for both plasma phospholipid fatty acids and cholesterol ester fatty acids), please provide analysis results for each of the biomarkers.

For the purposes of this analysis, the relationship of each fatty acid of interest with diabetes will be assessed:

1. using study-specific quintiles (primary)
2. continuously (% total fatty acids per 1 SD increment; for nested case-control studies, we will use the SD of the control group) (secondary)

Analysis Plan for Each Participating Study (study-specific analyses):

Baseline descriptive information will be collected (see Information to Be Collected section below for details on variables of interest). Additionally, we will assess the correlations of 16:0, c16:1n7, 18:0, and c18:1n9 with each other.

The association of each individual fatty acid of interest (16:0, c16:1n7, 18:0, and c18:1n9) with incident diabetes will be assessed both categorically (quintiles) and continuously (% total fatty acids per 1 SD increment) using multiple Cox regression models (for cohort studies) or conditional logistic regression (for case-control studies). Follow-up time will be calculated from baseline (time of fatty acid measurement) to date of development of incident diabetes, death from any cause, or loss to follow-up; participants will be censored at the time of diabetes diagnosis, death, or loss to follow-up.

We will examine the association of each fatty acid of interest with incident diabetes using results from three regression models that adjust for different sets of covariates. The primary model will adjust for potential confounders, including age, sex, field site (if necessary), socio-demographic variables (race, education, and occupation), health behaviors (physical activity, smoking, and alcohol use), family history of diabetes, menopausal status, exogenous hormone use, prevalent hypertension (treated or self-reported), prevalent dyslipidemia (treated or self-reported), prevalent coronary heart disease, and self-reported health status. A second model will additionally adjust for BMI and waist circumference. A third model (exploratory model) will further adjust for circulating 16:0 (for analysis of c16:1n7, 18:0, and c18:1n9) and triglycerides, to examine whether conversion from 16:0 to c16:1n7, 18:0, and c18:1n9 is associated with incident diabetes, independently of 16:0 (precursor) and triglycerides (end-product of lipogenesis).

We will also examine potential interaction of age (modeled continuously), sex, race, and BMI (modeled continuously) with each plasma phospholipid saturated fatty acid of interest (modeled continuously) on risk of incident diabetes. To evaluate interaction, a multiplicative interaction term for each factor of interest will be included in a model that also adjusts for age, sex, field site (if necessary), socio-demographic variables (race, education, and occupation), health behaviors (physical activity, smoking, and alcohol use), family history of diabetes, menopausal status, exogenous hormone use, prevalent hypertension (treated or self-reported), prevalent dyslipidemia (treated or self-reported), prevalent coronary heart disease, self-reported health status, BMI and waist-circumference.

Analysis Plan in Pooling Results:

Descriptive statistics will be tabulated across studies.

Meta-analyses will examine the relationship of each circulating fatty acid (16:0, c16:1n7, 18:0, and c18:1n9) with incident diabetes using inverse-variance-weighted fixed-effects models. We will also examine potential interaction of each circulating (16:0, c16:1n7, 18:0, and c18:1n9) with age, sex, race, and BMI on risk of diabetes by meta-analyzing the multiplicative interaction terms for each age, sex, race, or BMI with each fatty acid of interest on risk of incident diabetes.

Additionally, we will test for potential heterogeneity by country, fasting state, lipid fraction, and study design, by meta-regression and by stratified meta-analysis.

**INFORMATION TO BE REQUESTED:**

**(I) Descriptive information:**

We will request the following study-specific information. If the study is a nested case-control study, please provide descriptive information for the controls only.

- Baseline (year)
- End of follow-up (year)
- Total number of participants available for analysis
- Total number of participants excluded (i.e.,<18 years of age, prevalent diabetes)
- Total number of incident cases of diabetes
- Total number of person-years (if a nested case-control study, person-years of the source population will be requested)
- Circulating 16:0 (mean, median, SD, range)
- Circulating c16:1n7 (mean, median, SD, range)
- Circulating 18:0 (mean, median, SD, range)
- Circulating c18:1n9 (mean, median, SD, range)

If multiple lipid fractions were assessed, statistics of each fraction will be obtained.

- Sex (% female)
- Age (mean±SD)
- Race (study specific)
- Smoking (% never, %former, % current)
- Alcohol intake (drinks/day: mean±SD or grams/day: mean±SD)
- BMI (kg/m^2^) and waist circumference (cm) (mean±SD each)
- Prevalent hypertension, dyslipidemia, heart disease (% each)
- Menopausal status and hormone use among women (% in women for each)
- Family history of diabetes (%)
- Socioeconomic variables (e.g. categories of education and occupation, as defined by each study)
- Physical activity (hours/week: mean±SD or kcal/week: mean±SD or otherwise defined by the study)
- HbA1c (% of hemoglobin)
- Fasting glucose (mg/dL) (mean±SD)
- Fasting insulin (IU/mL) (mean±SD)
- HOMA-IR (mean±SD)
- HOMA-β (mean±SD)
- Triglycerides (mg/dL) (mean±SD)
- HDL cholesterol (mg/dL) (mean±SD)

(II) Methodological information

- Laboratory methods and relevant citation information for fatty acids assessment, including the tissue fraction of fatty acids measured.
- Fatty acid fractions
- Total number of fatty acids measured
- Laboratory methods and relevant citation information for fasting glucose assessment

(III) Correlations between fatty acids

- Pearson correlation matrix of levels of 16:0, c16:1n7, 18:0, and c18:1n9 (mean, median, SD, range).

For a nested case-control studies, please provide correlation matrix for controls

(IV) Main results (regression coefficients and robust standard errors, SE)

1. Analysis of 16:0, c16:1n7, 18:0, and c18:1n9 acids with incident diabetes (each variable modeled separately)

Model 1: adjusted for age, sex, field site (if necessary), race, education, occupation, physical activity, smoking, alcohol use, prevalent hypertension (treated or self-reported), prevalent dyslipidemia (treated or self-reported), prevalent coronary heart disease, and self-reported health status

Model 2: adjusted for all model 1 covariates, as well as BMI and waist circumference

Model 3: adjusted for all model 2 covariates, as well as triglycerides and circulating 16:0

For quintile analyses, please provide:

- regression coefficients + robust SE for each quintile (referent group=lowest quintile)
- number of diabetes cases in each quintile
- total n in each quintile
- total person-years in each quintile
- median level of circulating in each quintile

For continuous analyses (per SD), please provide:

- regression coefficients + robust SE

A spreadsheet (excel file) that details all requested information will be prepared and distributed to each participating cohort.

(V) Results for potential interaction

We will assess the interaction of each circulating levels of 16:0, c16:1n7, 18:0, and c18:1n9 with

(1) age (modeled continuously)

(2) sex (male, female)

(3) race (using cohort-specific dummy variables)

(4) BMI (modeled continuously)

on risk of diabetes to examine potential heterogeneity.

(1) Analyses include a multiplicative (cross-product) interaction term for each of 16:0, c16:1n7, 18:0, and c18:1n9 with age, sex, race or BMI (modeled separately) in a model also adjusted for age, sex, field site (if necessary), socio-demographic variables (race, education, and occupation), health behaviors (physical activity, smoking, and alcohol use), family history of diabetes, menopausal status, exogenous hormone use, prevalent hypertension (treated or self-reported), prevalent dyslipidemia (treated or self-reported), prevalent coronary heart disease, self-reported health status, BMI and waist-circumference.

For each interaction analysis, please provide:

- regression coefficient + robust SE the cross-product interaction term

A spreadsheet (excel file) that details all requested information for the interaction analyses will be prepared and distributed to each participating cohort. If a significant interaction is observed by pooling study-specific interaction terms in fixed-effect meta-analyses, stratified analyses of the factor of interest will be requested after post-hoc decision of cut points for stratification.

SUMMARY

Roles of *de novo* lipogenesis in diabetes etiology are not systematically evaluated by long-term prospective epidemiologic studies. We will assess the association of circulating levels of fatty acid biomarkers of *de novo* lipogenesis with incident diabetes in the CHARGE consortium.

**REFERENCES**

1. Ameer F, Scandiuzzi L, Hasnain S, Kalbacher H, Zaidi N. De novo lipogenesis in health and disease. *Metabolism*. 2014.

2. Lodhi IJ, Wei X, Semenkovich CF. Lipoexpediency: de novo lipogenesis as a metabolic signal transmitter. *Trends Endocrinol Metab*. 2011;22(1):1–8.

3. Borkman M, Storlien LH, Pan DA, Jenkins AB, Chisholm DJ, Campbell L V. The relation between insulin sensitivity and the fatty-acid composition of skeletal-muscle phospholipids. *New Engl J Med*. 1993;328(4):238–44.

4. International Diabetes Federatoin. The Global Burden. In: *IDF Diabetes Atlas*. 6th ed. Brussels, Belgium: International Diabetes Federation; 2013:29–49.

5. Maedler K, Spinas GA, Dyntar D, Moritz W, Kaiser N, Donath MY. Distinct effects of saturated and monounsaturated fatty acids on beta-cell turnover and function. *Diabetes*. 2001;50(1):69–76.

6. Maedler K, Oberholzer J, Bucher P, Spinas GA, Donath MY. Monounsaturated fatty acids prevent the deleterious effects of palmitate and high glucose on human pancreatic beta-cell turnover and function. *Diabetes*. 2003;52(3):726–733.

7. Cnop M, Hannaert JC, Hoorens A, Eizirik DL, Pipeleers DG. Inverse relationship between cytotoxicity of free fatty acids in pancreatic islet cells and cellular triglyceride accumulation. *Diabetes*. 2001;50(8):1771–1777.

8. Shi H, Kokoeva M V, Inouye K, Tzameli I, Yin H, Flier JS. TLR4 links innate immunity and fatty acid – induced insulin resistance. *J Clin Invest*. 2006;116(11):3015–3025.

9. King IB, Lemaitre RN, Kestin M. Effect of a low-fat diet on fatty acid composition in red cells, plasma phospholipids, and cholesterol esters: investigation of a biomarker of total fat intake. *Am J Clin Nutr*. 2006;83(2):227–36.

10. Wu JHY, Lemaitre RN, Imamura F, et al. Fatty acids in the de novo lipogenesis pathway and risk of coronary heart disease: the Cardiovascular Health Study. *Am J Cardiol*. 2011;94(2):431–438.

11. Siler SQ, Neese RA, Hellerstein MK. De novo lipogenesis, lipid kinetics, and whole-body lipid balances in humans after acute alcohol consumption. *Am J Clin Nutr*. 1999;70(5):928–36.

12. Hudgins LC, Hellerstein M, Seidman C, Neese R, Diakun J, Hirsch J. Human fatty acid synthesis is stimulated by a eucaloric low fat, high carbohydrate diet. *J Clin Invest*. 1996;97(9):2081–2091.

13. Krachler B, Norberg M, Eriksson JW, et al. Fatty acid profile of the erythrocyte membrane preceding development of Type 2 diabetes mellitus. *Nutr Metab Cardiovasc Dis*. 2008;18(7):503–510.

14. Patel PS, Sharp SJ, Jansen E, et al. Fatty acids measured in plasma and erythrocyte-membrane phospholipids and derived by food-frequency questionnaire and the risk of new-onset type 2 diabetes: a pilot study in the European Prospective Investigation into Cancer and Nutrition (EPIC)–Norfolk. *Am J Clin Nutr*. 2010;92(5):1214–1222.

15. Kröger J, Zietemann V, Enzenbach C, et al. Erythrocyte membrane phospholipid fatty acids, desaturase activity, and dietary fatty acids in relation to risk of type 2 diabetes in the European Prospective Investigation into Cancer and Nutrition (EPIC) - Potsdam Study. *Am J Clin Nutr*. 2010;93(1):127–142.

16. Hodge AM, English DR, O’Dea K, et al. Plasma phospholipid and dietary fatty acids as predictors of type 2 diabetes: interpreting the role of linoleic acid. *Am J Clin Nutr*. 2007;86(1):189–97.

17. Wang L, Folsom AR, Zheng Z-JJ, Pankow JS, Eckfeldt JH. Plasma fatty acid composition and incidence of diabetes in middle-aged adults: the Atherosclerosis Risk in Communities (ARIC) Study. *Am J Clin Nutr*. 2003;78(1):91–8.
